# A Continuous Battle for Host-Derived Glycans Between a Mucus Specialist and a Glycan Generalist *in vitro* and *in vivo*

**DOI:** 10.3389/fmicb.2021.632454

**Published:** 2021-06-24

**Authors:** Ioannis Kostopoulos, Steven Aalvink, Petia Kovatcheva-Datchary, Bart Nijsse, Fredrik Bäckhed, Jan Knol, Willem M. de Vos, Clara Belzer

**Affiliations:** ^1^Laboratory of Microbiology, Wageningen University, Wageningen, Netherlands; ^2^Wallenberg Laboratory, Department of Molecular and Clinical Medicine, University of Gothenburg, Gothenburg, Sweden; ^3^Laboratory of Systems and Synthetic Biology, Wageningen University, Wageningen, Netherlands; ^4^Danone Nutricia Research, Utrecht, Netherlands; ^5^Human Microbiome Research Program, Faculty of Medicine, University of Helsinki, Helsinki, Finland

**Keywords:** *Akkermansia muciniphila*, *Bacteroides thetaiotaomicron*, mucin glycans, glycosyl hydrolases, antimicrobial proteins, germ free mice

## Abstract

The human gastrointestinal tract is colonized by a diverse microbial community, which plays a crucial role in human health. In the gut, a protective mucus layer that consists of glycan structures separates the bacteria from the host epithelial cells. These host-derived glycans are utilized by bacteria that have adapted to this specific compound in the gastrointestinal tract. Our study investigated the close interaction between two distinct gut microbiota members known to use mucus glycans, the generalist *Bacteroides thetaiotaomicron* and the specialist *Akkermansia muciniphila in vitro* and *in vivo*. The *in vitro* study, in which mucin was the only nutrient source, indicated that *B. thetaiotaomicron* significantly upregulated genes coding for Glycoside Hydrolases (GHs) and mucin degradation activity when cultured in the presence of *A. muciniphila*. Furthermore, *B. thetaiotaomicron* significantly upregulated the expression of a gene encoding for membrane attack complex/perforin (MACPF) domain in co-culture. The transcriptome analysis also indicated that *A. muciniphila* was less affected by the environmental changes and was able to sustain its abundance in the presence of *B. thetaiotaomicron* while increasing the expression of LPS core biosynthesis activity encoding genes (*O-*antigen ligase, Lipid A and Glycosyl transferases) as well as ABC transporters. Using germ-free mice colonized with *B. thetaiotaomicron* and/or *A. muciniphila*, we observed a more general glycan degrading profile in *B*. *thetaiotaomicron* while the expression profile of *A. muciniphila* was not significantly affected when colonizing together, indicating that two different nutritional niches were established in mice gut. Thus, our results indicate that a mucin degrading generalist adapts to its changing environment, depending on available carbohydrates while a mucin degrading specialist adapts by coping with competing microorganism through upregulation of defense related genes.

## Introduction

The gut mucosal layer is the first line of defense against damage and infection (Corfield, 2015; Bergstrom et al., 2020). At the same time, mucin glycans serve as a consistent nutrient foundation for members of the gut microbiota (Martens et al., 2008). Mucins consist predominantly of carbohydrates, mainly *N-*acetylgalactosamine (GalNAc), *N-*acetylglucosamine (GlcNAc), fucose (fuc), galactose (gal), and sialic acid (Neu5ac) (Bansil and Turner, 2006; Abodinar et al., 2016). In mucus, core-glycans are covalently linked to serine or threonine residues to form complex *O-*linked glycan structures (Bergstrom and Xia, 2013). The type of *O-*glycans produced in the human intestinal tract depends on many factors encoded within the human genome, including the expression of genes responsible for mucin synthesis (MUC) (Robbe et al., 2004). The glycan diversity is provided by the peripheral epitopes (Tailford et al., 2015). For example, the α1,2-fucosylation of core glycan structures is found in populations that carry the secretor gene (Mollicone et al., 1985). The fucosyltransferase 2 (FUT2) gene is responsible for the synthesis of type 1 H antigens, whose secretion determines the expression of the ABH and Lewis histo-blood antigens in the intestinal mucosa (Wacklin et al., 2014). Moreover, the expression of FUT2 gene contributes to the microbiota composition (Wacklin et al., 2011; Kashyap et al., 2013; Garrido et al., 2015). Bacteria that can degrade mucin glycans can colonize the mucosal surface and become core species in the gastrointestinal (GI) tract (Bergstrom and Xia, 2013; Tailford et al., 2015).

Several gut bacteria species are capable of degrading these host substrates (Salyers et al., 1977). For example, the ability of *Bacteroides* species to utilize diverse glycans depends on a series of gene clusters, which are termed the Polysaccharide Utilization Loci (PULs) (Bjursell et al., 2006). PULs encode cell envelope systems that typically include glycolytic enzymes and the homologs of two outer membrane proteins (SusC and SusD) that are part of the first described PUL, the starch utilization system (Sus) locus (Martens et al., 2009). One well-studied glycan-degrading bacterium is *Bacteroides thetaiotaomicron* VPI-5482, which has 88 PULs and 221 mucin-degrading enzymes (Martens et al., 2008). *B. thetaiotaomicron* is a prominent member of the human gut microbiota capable of growing on many different plant and host glycans (Salyers et al., 1977; Martens et al., 2008). When *B. thetaiotaomicron* colonizes the distal gut of adult germ-free mice maintained on a diet rich in plant glycans, it increases the expression of genes involved in the catabolism of dietary substrates (Sonnenburg, 2005). In contrast, in adult mice fed a diet devoid of complex glycans, *B. thetaiotaomicron* alters its response to express genes involved in targeting host glycans (Bjursell et al., 2006). Transcriptomic analyses highlighted specific polysaccharide-utilization loci (PULs) including genes coding for putative glycoside hydrolases (GHs), such as α-L-fucosidase, endo-β-N-acetylglucosaminidase, endo-β-galactosidase and α-mannosidase, which were upregulated when *B. thetaiotaomicron* was grown on mucin O-glycans or in mono-associated mice—when compared to *in vitro* glucose control (Martens et al., 2008, 2011).

Another well-studied mucin degrader, *Akkermansia muciniphila* ATCC BAA-835, belongs to Verrucomicrobium phylum and is also a prominent member of the human microbiota. *A. muciniphila* is associated with a healthy mucosal layer, stimulates mucus thickness and barrier function (Reunanen et al., 2015; Van Der Lugt et al., 2019) and metabolic state as it has been inversely correlated with obesity (Karlsson et al., 2012; Dao et al., 2016), metabolic diseases (Type 2 diabetes) (Zhang et al., 2013) as well as intestinal disorders [inflammatory bowel disease (IBD) and appendicitis] (Png et al., 2010; Swidsinski et al., 2011; Rajilić-Stojanović et al., 2013). Depommier et al. illustrated that supplementation with *A. muciniphila* in overweight and obese humans reduced the levels of blood markers related to liver dysfunction and inflammation, while the overall gut microbiome remained unaffected (Depommier et al., 2019). *A. muciniphila* is a mucin degrading specialist that possesses 61 (~3% of its relatively small genome) mucin degrading enzymes to utilize mucus glycans as the sole carbon, energy and nitrogen source (Collado et al., 2007).

Both *B. thetaiotaomicron* and *A. muciniphila* belong to dominant phyla in the adult human gut (Eckburg et al., 2005). In a complex environment, such as the human gut microbiome, the search for nutrients is vital for the survival of the gut residents (Bauer et al., 2018). Bacterial communities exist under continuous competition and collaborative efforts of the bacteria in them. Many different types of interactions occur within a complex microbial ecological network. In general, two mechanisms of competition exist: (i) exploitative competition, where members compete for shared nutrients and resources and (ii) interference competition, in which a member directly harms a competitor, often through the production of antimicrobial molecules (Ghoul and Mitri, 2016).

To investigate the microbe-to-microbe interaction under the continuous influx of host-derived glycans (mucin), we developed an *in vitro* bioreactor model with a mucin-glycan specialist (*A. muciniphila)* and a glycan generalist (*B. thetaiotaomicron)*. Mono-associated bioreactors were inoculated and used as control. Host-microbe interactions were evaluated through a gnotobiotic mouse model colonized with the same set of microbes.

## Results

### A Stable Co-culture *in vitro* Leads to the Adaptation of Glycan Degradative Pathways

**Figure 1:**
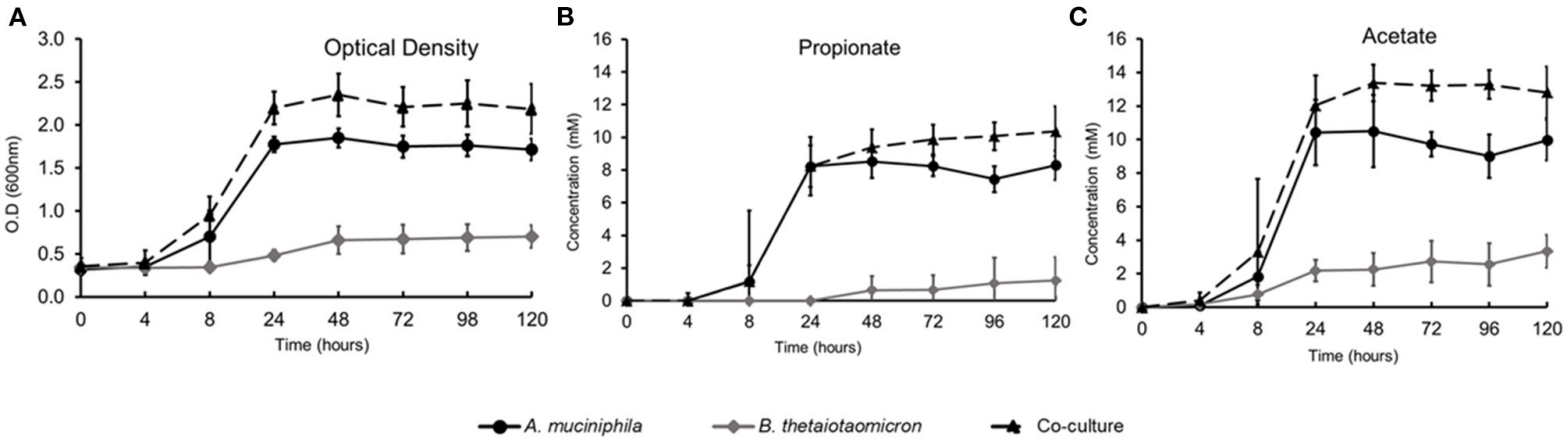
Growth and SCFAs production during *in vitro* fermentation. **(A)** Optical Density (O.D.) measurements at 600 nm, **(B)** Propionate production, **(C)** Acetate production.

We established monocultures and a co-culture of *A. muciniphila* ATCC BAA-835 and *B. thetaiotaomicron* VPI-5482 in bioreactors with a continuous feed of mucin glycans. The bioreactors were sampled for measurements of metabolite production and total mRNA at the end of fermentation (120 h). The co-culture was characterized by higher growth and metabolic profile compared to the mono-associated bioreactors (Figures 1A–C). The total cell counts at the co-culture in the end of the fermentation (120 h) were 9.78 × 10^7^ cells/ml and 2.12 × 10^6^ cell/ml for *A. muciniphila* and *B. thetaiotaomicron*, respectively (Supplementary Table 1). *A. muciniphila* and *B. thetaiotaomicron* co-culture showed a significantly higher production (*q* < 0.05) of acetate and propionate compared to their monocultures. Moreover, as expected, 1,2-propanediol and succinate were also observed during the fermentation period, indicating continued fermentation of the monosaccharides released from the mucus glycans degradation. The amounts of SCFAs in the different conditions are shown in Supplementary Table 2.

We first focused on the adaptation of *A. muciniphila* and *B. thetaiotaomicron* to use their glycan degradation machinery. We measured expression of the genes from *B. thetaiotaomicron* and *A. muciniphila* in the community involved in carbohydrate metabolism and glycan biosynthesis and metabolism. The transcriptome data indicated that 306 genes from *B. thetaiotaomicron* involved in carbohydrate metabolism and glycan biosynthesis and metabolism were significantly different in the mono- and co-culture conditions. A total of 4% of these genes were significantly upregulated in the co-culture (*q* < 0.05). *B. thetaiotaomicron* expressed 218 of the 220-known mucin-degrading genes in both conditions. In the co-culture, *B. thetaiotaomicron* overexpressed (above 7.0-fold change) two genes coding for d-glycero-d-manno-heptose 1-phosphate kinase (BT_0474) and phosphoheptose isomerase (BT_0475) activity, respectively. Both genes were predicted to be involved in lipopolysaccharide biosynthesis. The capacity of *B. thetaiotaomicron* to utilize different complex dietary oligosaccharides relies on 88 polysaccharide utilization loci (PULs), which encompasses 18% of its genome (Martens et al., 2008). Therefore, we analyzed the changes in the PUL expression and compared the mRNA levels between mono- and co-culture conditions. In the co-culture, *B. thetaiotaomicron* significantly upregulated 93 genes belonging to 40 different PULs, while 77 genes associated with 22 PULs were downregulated. In the co-culture, there was significant upregulation of 40 different PULs that are described as being induced by host *O*-glycans. Three enriched PULs in the co-culture, encompassed within BT_0317-18, BT_2170-73, and BT_4038-40, had more than a 2.0-fold change. Two downregulated PULs, encompassed within BT_4652-62 (heparin sulfate) and BT_3324-49 (chondroitin sulfate) were significantly expressed in the monoculture (Supplementary Table 3).

In contrast, *A. muciniphila*, showed 143 genes, differentially expressed between mono- and co-culture, involved in carbohydrate metabolism and glycan biosynthesis and metabolism, and 5% of these genes were significantly upregulated and 2% were downregulated in the co-culture (Supplementary Table 3). In the co-culture, *A. muciniphila* significantly upregulated two lipopolysaccharide biosynthesis genes encoding for *O-*antigen ligase domain-containing protein (Amuc_0088) and lipid-A biosynthesis acyltransferase protein (Amuc_1541) (>2.0 fold-change, *q* < 0.05). From the 53 mucin-degrading genes found in our *A. muciniphila* transcriptome data, seven were significantly upregulated in the co-culture (>2.0 fold-change) (Supplementary Table 4).

### A Stable Co-culture *in vitro* Leads to the Altered Overall Expression of Carbohydrate-Active Enzyme Genes

**Figure 2:**
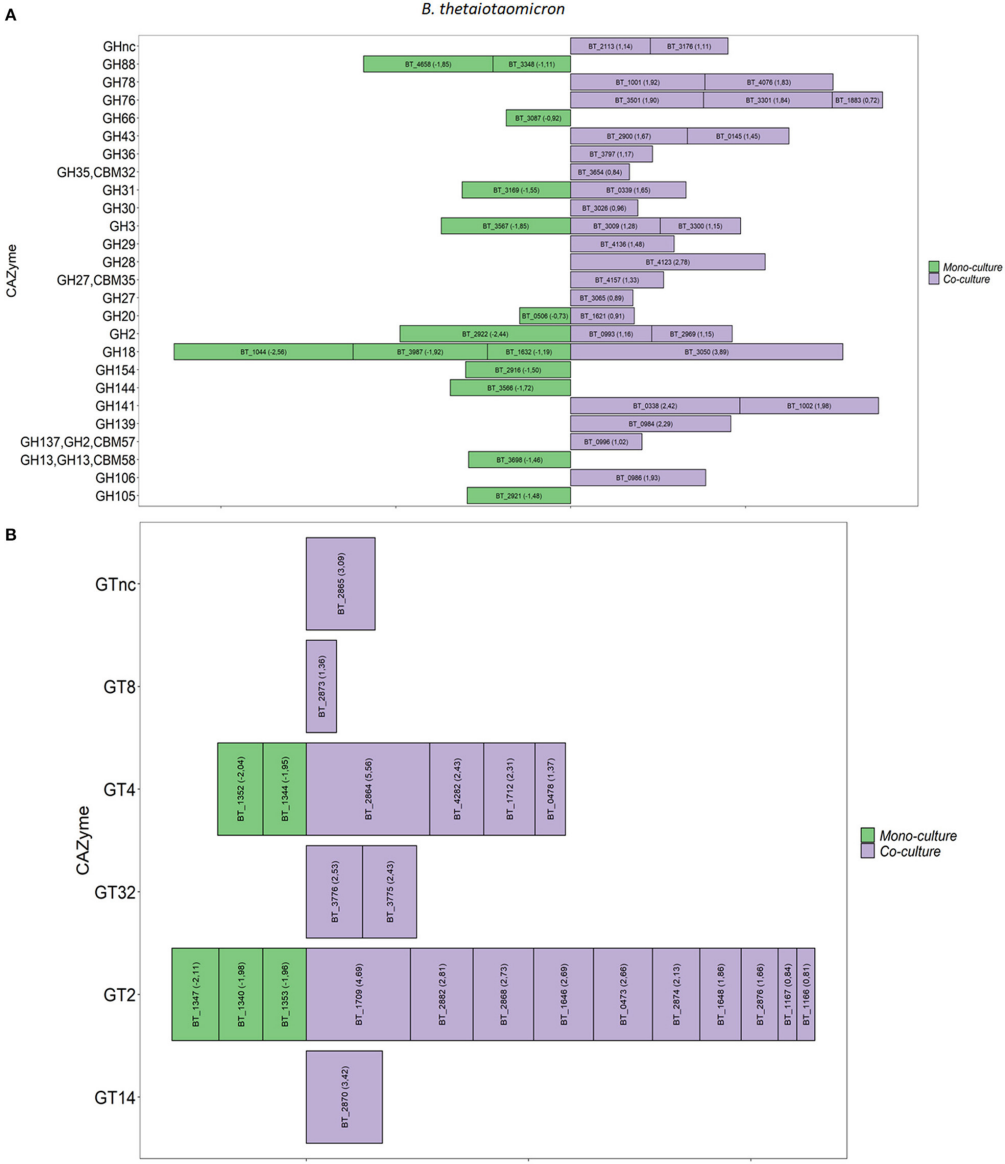
Differential expression of GHs and GTs between mono and co-culture during *in vitro* fermentation. **(A)** *B. thetaiotaomicron* GHs, **(B)** *B. thetaiotaomicron* GTs. Only the CAZymes with adjusted *p* < 0.05 and Log2 Fold Change ≥ 0.58 are shown as averages. The size of each bar is proportional to the Log2 Fold change (the number between brackets within each bar).

The overall expression of the CAZymes indicates that both bacteria are expanding their carbohydrate metabolic gene response in the co-culture. The genes encoding for carbohydrate-active enzymes (CAZymes) in the co-culture compared to monoculture were analyzed in detail. *B. thetaiotaomicron* altered its hydrolytic machinery between the two conditions. A significant increase in gene expression was detected for β-glucosidases (GH3, GH30), α-glucosidase (GH31), β-galactosidases (GH2, GH137), α-galactosidase (GH27, GH36), α-L-arabinosidase (GH43), α-L-fucosidases (GH29, GH139, GH141), α-mannanases (GH76), and α-L-rhamnosidases (GH78) in the co-culture (Figure 2A). We also observed that, in the co-culture, *B. thetaiotaomicron* expressed genes that could target different glycosidic linkages present in mucin. For example, four α-L fucosidases exhibited more than a 2.8-fold difference in the co-culture compared to the monoculture. Namely, BT_0984 and BT_4136 are predicted to have α1-2-O-Me-L-fucosidase and a1-3/1-4-fucosidase activity, respectively. BT_0338 and BT_1002 genes are classified as GH141 having α-L-fucosidase activity. Moreover, *B. thetaiotaomicron* showed an increase in the overall expression of GHs families in the co-culture (Supplementary Figure 1). Also, polysaccharides lyases (PL1, P10, PL27), encoding for pectate lyase, were enriched in the co-culture. Furthermore, in the co-culture, *B. thetaiotaomicron* significantly expressed more glycosyl transferases (GTs) from different families (> 1.8-fold change, *q* < 0.05), with GT2 (8 genes) and GT4 (3 genes) being the most representative GT families in the co-culture (Figure 2B). Interestingly, BT_1709 (GT2) and BT_2864 (GT4) displayed a 29.0 and 39.0-fold change, respectively.

In the co-culture, *A. muciniphila* showed a significant increase of three GH families that are predicted to be involved in the glycan degradation pathway. Namely, β-galactosidases (GH2), β-N-acetylhexosaminidases (GH20), and α-L-fucosidases (GH29) were significantly enriched in the co-culture. Furthermore, two families of GTs (GT2 and GT4) showed the highest increase of their overall expression in the co-culture conditions. Specifically, *A. muciniphila* significantly upregulated four genes encoding GT2 (Amuc_2094) and GT4 (Amuc_2088, Amuc_2089 and Amuc_2090) (1.9-fold change, *q* < 0.05; Supplementary Figures 2A,B). The list of *A. muciniphila* and *B. thetaiotaomicron* CAZymes found to be upregulated in the co-culture is summarized in Supplementary Tables 5, 6, respectively.

### *In vitro* Microbe-To-Microbe Interaction Between *B. thetaiotaomicron* and *A. muciniphila*

**Figure 3:**
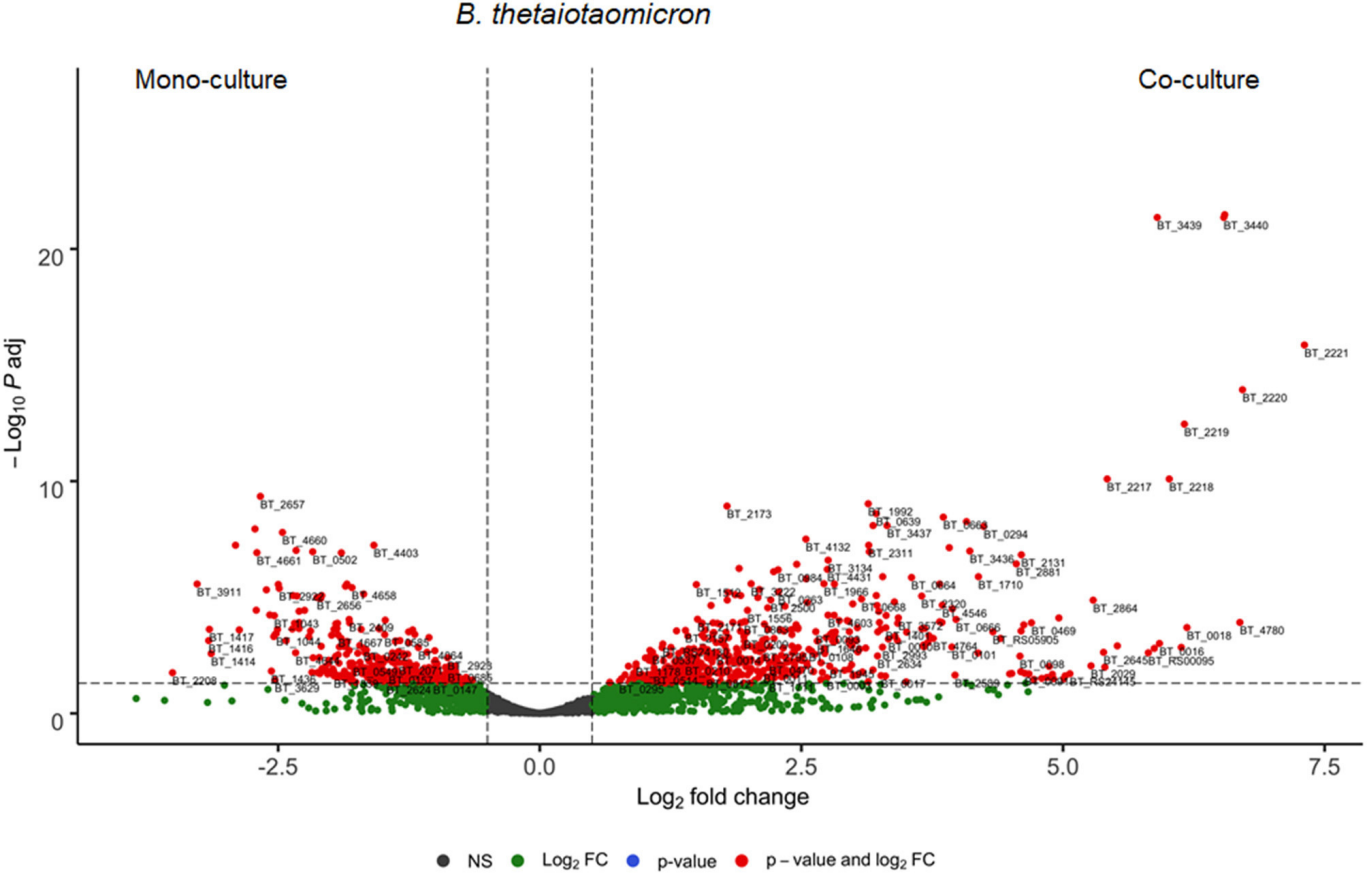
Differential expressed genes of *B. thetaiotaomicron* between mono- and co-culture during *in vitro* fermentation. Volcano plot of the differential expressed *B. thetaiotaomicron* genes of mono- vs. co-culture. Positive Log2 fold change indicates upregulation in co-culture. The horizontal dashed black line adjusted *p*-value threshold (*q*-value = 0.05). The vertical dashed black lines indicate Log2 fold change threshold (Log2 fold change = 0.58).

**Figure 4:**
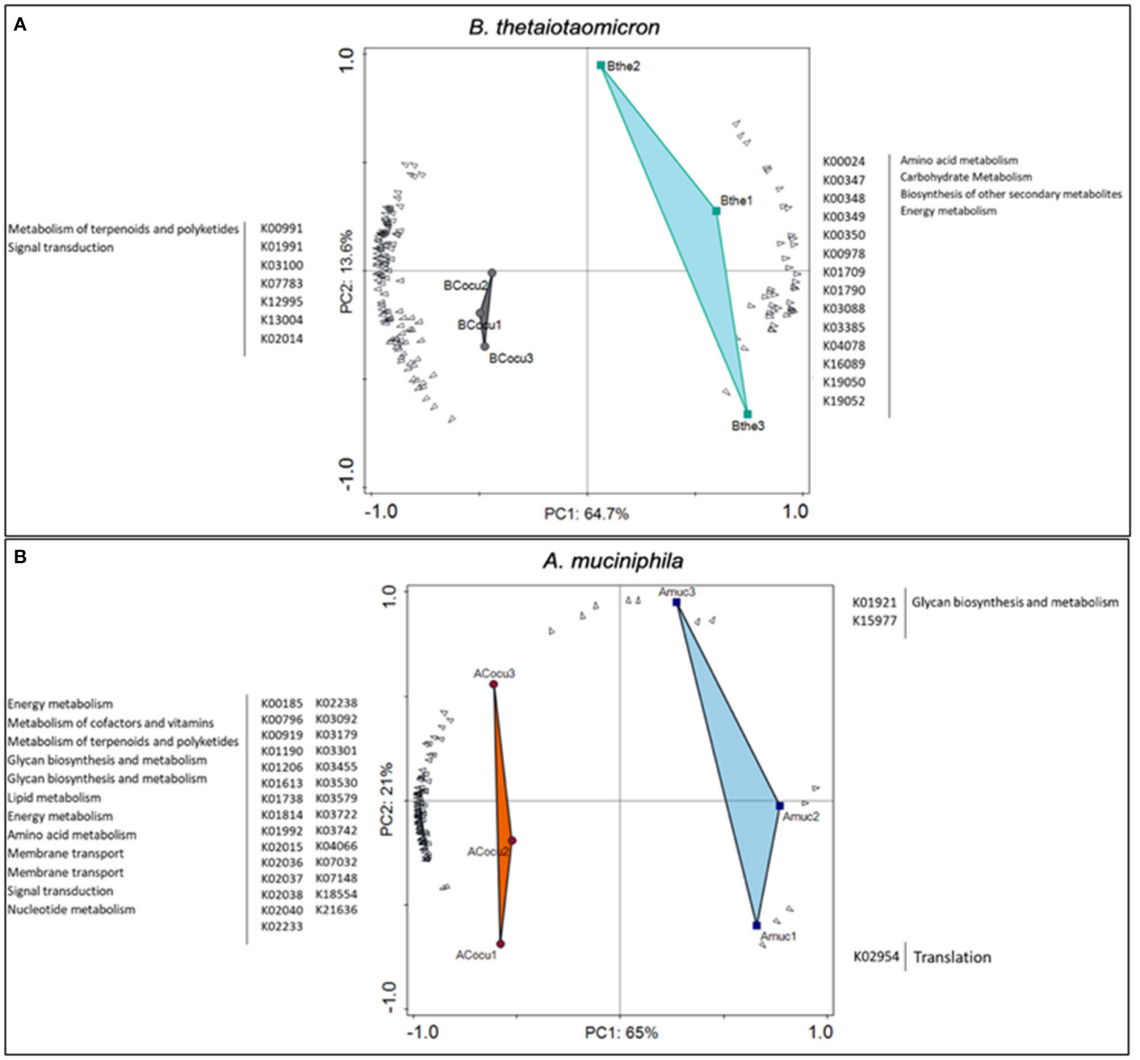
Differential expressed genes and KEGG Orthologies (KOs) of *B. thetaiotaomicron* and *A. muciniphila* between mono- and co-culture during *in vitro* fermentation. **(A)** Principal Component analysis (PCA) showing the most variable KOs between *B. thetaiotaomicron* mono- and co-culture. **(B)** Principal Component analysis (PCA) showing the most variable KOs between *A. muciniphila* mono- and co-culture.

Thereafter, we analyzed genes involved in the interaction between *B. thetaiotaomicron* and *A. muciniphila*. We identified in *B. thetaiotaomicron*'s transcriptome, 1012 differentially expressed genes, of which 597 genes (59%) were upregulated in the co-culture and 415 genes (41%) were downregulated (Figure 3). In the co-culture, *B. thetaiotaomicron* significantly upregulated two different operons (BT_2217–2221 and BT_3439–3441) of hypothetical proteins that exhibit the highest fold change compared to the monoculture. BT_2217-2221 exhibited between 30 and 120-fold change (*q* < 0.05). The other operon (BT_3439–3441) exhibited between 60 and 96-fold change (*q* < 0.05). The latter cluster contains a BT_3439 gene that encodes for the Membrane Attack Complex/Perforin (MACPF) domain-containing protein. Furthermore, the metabolism of terpenoids and polyketides (K00919, K00991) and the signal transduction (K03100, K07783, K07713) were the most variable metabolic processes in the co-culture (Figure 4A).

**Figure 5:**
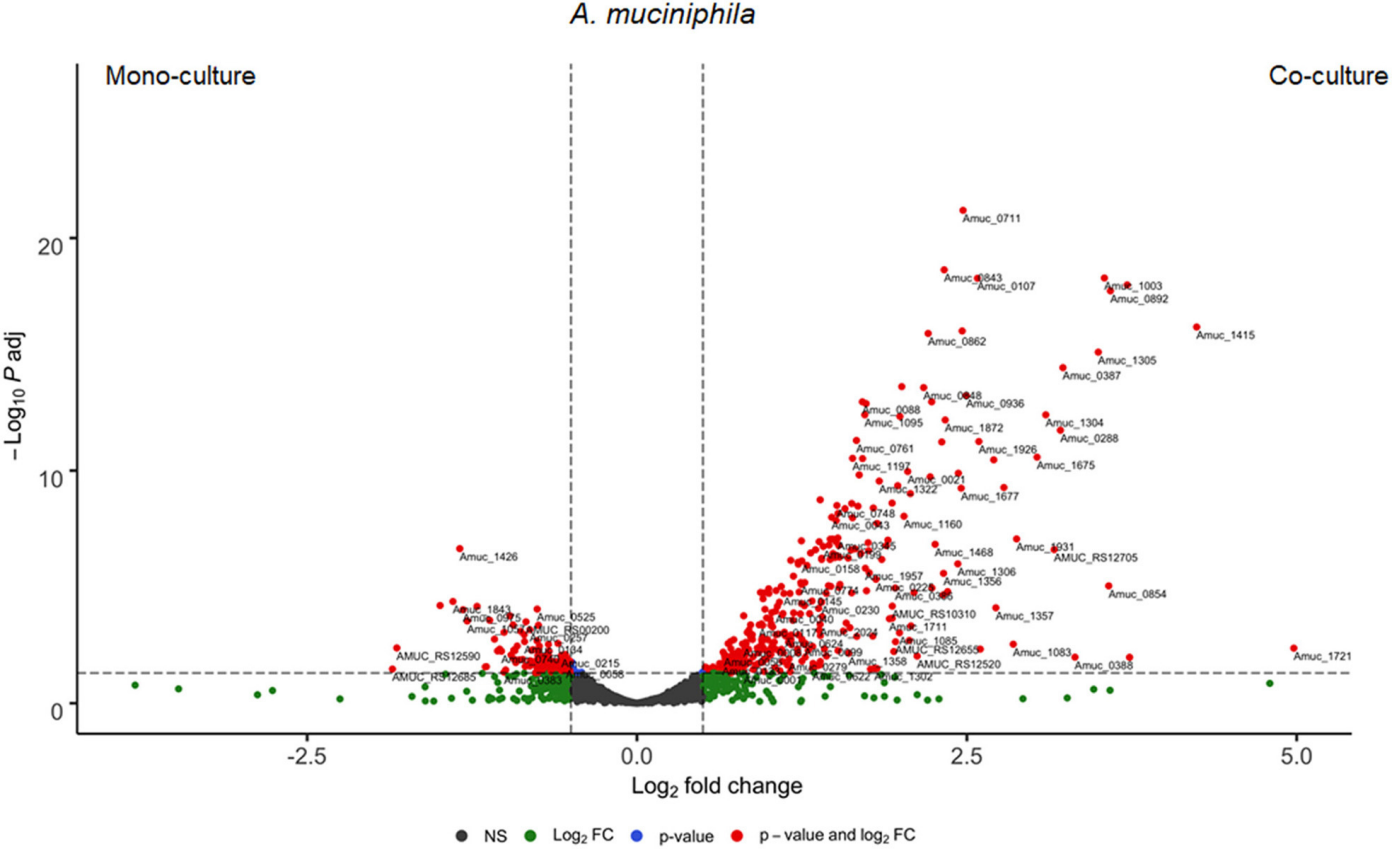
Differential expressed genes of *A. muciniphila* between mono- and co-culture during *in vitro* fermentation. Volcano plot of the differential expressed *A. muciniphila* genes of mono- vs. co-culture. Positive Log2 fold change indicates upregulation in co-culture. The horizontal dashed black line adjusted *p*-value threshold (*q* = 0.05). The vertical dashed black lines indicate Log2 fold change threshold (Log2 fold change = 0.58).

*A. muciniphila* differentially regulated 454 genes, in which 347 genes (76%) were upregulated and the rest 107 genes (24%) were downregulated in the co-culture (Figure 5). Interestingly, 7% of the significantly upregulated *A. muciniphila* genes encoded for transporter activity participate in membrane transport, signal transduction and/or signaling and cellular processes pathways. In the co-culture experiment, *A. muciniphila* demonstrated the highest fold difference (>4.0-fold change, *q* < 0.05) of a gene cluster that consists of two ABC transporter permeases (Amuc_1304 and Amuc_1305), two phosphate-binding proteins (Amuc_1304 and Amuc_1306) and is found to be part of the same gene operon (Amuc_1295–1306). In addition, three genes that belong to the same operon and encode for transporter permease (Amuc_1379 and Amuc_1380) and ATP-binding protein (Amuc_1381) activity, exhibited more than a 2.0-fold difference in the co-culture. Additionally, three membrane transport genes from the same operon (Amuc_0143-45) were predicted to be involved in the oligopeptide transport system. These genes that encode for oligopeptide substrate-binding (Amuc_0145) and permease (Amuc_0143, Amuc_0144) protein activity exhibited above a 1.5-fold difference in the co-culture conditions (*q* < 0.05). Furthermore, the transcriptome analysis revealed a significant upregulation (>1.5-fold change, *q* < 0.05) of genes that were involved in a two-component system pathway (Amuc_0021, Amuc_0828 and Amuc_0831, Amuc_0889, and Amuc_1151 – 1152). The 50 most variable and the most significantly upregulated genes from both members of the community are illustrated in Supplementary Tables 7, 8.

### *In vivo* Co-colonization of *A. muciniphila* and *B. thetaiotaomicron* in Germ-Free Mice

Thereafter, we investigated the mono- and co-culture conditions in a gnotobiotic mouse model. Samples from the cecum and distal colon were taken for the quantification of the bacterial counts as well as the total RNA extraction and transcriptome data were generated. The transcriptome analysis of *B. thetaiotaomicron* in the *in vivo* conditions showed no significant differences in the regulation of host glycan-degrading genes or the PULs between mono-colonization and co-colonization.

During the 14 days of colonization period, the mice received a standard diet rich in dietary fibers (chow diet). In the end of the colonization period, we showed that both bacteria colonized both cecum and distal colon of mice. *B. thetaiotaomicron* was found to be more abundant than *A. muciniphila* in both cecum (1.10 × 10^5^ ± 3.74 × 10^4^ cells/ng of DNA) and distal colon (1.24 × 10^5^ ± 6.30 × 10^3^ cells/ng of DNA) in the co-culture (Supplementary Table 1). The transcriptome analysis of *A. muciniphila* in cecum revealed that 185 genes were significantly upregulated, and 116 genes were downregulated in the co-colonized mice (Supplementary Figure 3), while no significant differences were observed in distal colon between mono- and co-colonized mice. *A. muciniphila* in cecum in the co-colonization experiment showed significant differences in the expression profile of genes involved in carbohydrate metabolism and glycan biosynthesis and metabolism. Co-colonization conditions in cecum showed significant upregulation (above 2.0 and up to a 7.0-fold change) of the genes involved in two-component system pathways (Amuc_0827 and Amuc_0831 and Amuc_1151–1152) for *A. muciniphila*. Furthermore, 17 genes within the operon Amuc_2077–Amuc_2097 (Amuc_2078-79, Amuc_2081-94, and Amuc_2096) were differentially expressed, which demonstrated more than a 1.6-fold change in the co-colonized mice. Interestingly, nine genes of this operon (45%) were found to encode for glycosyl transferase activity (GT2 and GT4). On the other hand, fourteen genes involved in carbohydrate metabolism and four genes participating in glycan biosynthesis and metabolism were found to be significantly downregulated in the co-colonized mice.

### Host Response Toward Mono- and Co-cultures Lead to a Differential Response in Immune and Gene Expression

Host response toward the colonization with mono- and co-cultures of *B. thetaiotaomicron* and *A. muciniphila* was monitored by total RNA-sequencing of the cecum and distal colon. The transcriptional profile showed no significant differences in mouse cecum response between mono-colonization with *B. thetaiotaomicron* and co-colonization. In the distal colon, the comparison of *B. thetaiotaomicron* mono-associated mice with the co-associated revealed 228 significantly upregulated genes in the co-colonized mice. The 10 most significantly impacted genes in the co-colonized mice (Tgm3, Atp12a, Cd74, Lyz2, Slc40a1, B3gnt7, Pag1, Ptprc, Bach2, Lrrk2) exhibited more than a 2.0-fold difference. Next, six genes showed more than a 10-fold difference in the co-associated mice (St8sia5, Glycam1, Atp12a, Spink5, Nr1h5, Casp14) (Supplementary Table 9). Finally, the co-colonized mice overexpressed three genes coding for glycosyltransferase activity and involved in glycan biosynthesis (B3gnt7, Chst3, Gal3st2c, St8sia5). Specifically, B3gnt7 (β-1,3-N-acetylglucosaminyltransferase) and Chst3 (chondroitin 6-sulfotransferase) participate in the glycosaminoglycan biosynthesis pathway, while St8sia5 (α-N-acetyl-neuraminide a-2,8-sialyltransferase) and Gal3st2c (galactose-3-O-sulfotransferase) are involved in glycosphingolipid biosynthesis and unclassified metabolism pathways, respectively. A trend toward increased expression of genes coding for protein glycosylation activity (B3gat1, Gal3st2, Gal3st2b) was observed in mice colonized with both organisms. In both the cecum and the distal colon, no significant differences were observed in the expression of mucin genes between mono- and co-colonization.

The transcriptional profile showed 23 genes significantly affected by the presence of glycan degraders together in mouse cecum compared to *A. muciniphila* associated mice (Supplementary Table 8). The 10 most significantly abundant genes in the co-colonized mice (Lgals3, Aqp8, Sdc1, Adipor2, Slc2a1, Prdx5, Tsku, Tmem171, Cgref1, Kyat1) exhibited more than a 1.5-fold difference. Five genes (Zzef1, Cckar, Zfp987, Epha6, Gjb4) exhibited more than a 10-fold difference in the co-culture. Zzef1 and Zfp987 genes encoding for zinc finger protein activity were 1.10 × 10^7^- and 112-fold different in the co-colonization, respectively. Furthermore, we observed that an acetyl-Coenzyme A (Acaa1b) encoding gene was significantly overexpressed in the co-associated mice (2.65-fold change, *q* < 0.05). Additionally, genes encoding for protein glycosylation activity were upregulated non-significantly in the co-colonized mice. No significant differences were noticed in the expression of cecal mucin genes in the mice colonized with the two bacteria. In the distal colon, no significant differences in the mice's responses between mono-colonization with *A. muciniphila* and co-colonization were found. In the distal colon, though, a higher expression of mucin genes (Muc2, Muc3a, Muc4, Muc6, Muc16, Muc20) was detected in the co-colonization study compared to mono-colonization.

## Discussion

This study investigated the interaction between a mucus-degrading specialist, *A. muciniphila* and a mucin-degrading generalist *B. thetaiotaomicron* during growth *in vitro* and *in vivo* conditions. The results demonstrated that these two glycan-degrading bacteria reached a stable state over time *in vitro* where both species co-exist and are metabolically active. Furthermore, the transcriptional response of both bacteria was altered when they were cultured together in either a lab bioreactor or animal gut.

In the *in vitro* co-culture, *B. thetaiotaomicron* showed increased expression of its hydrolytic machinery. A possible explanation for this might be that *B. thetaiotaomicron* in the presence of *A. muciniphila* attempts to reach the mucin glycans by deploying as many GHs as possible to gain a competitive advantage. Another possible explanation is that *B. thetaiotaomicron* attempts to reduce *A. muciniphila*'s fitness in the community. The latter is often described as an exploitative competition where a member of the community restricts the competitor's access to nutrients via the secretion of digestive enzymes (Diggle et al., 2007; Rendueles and Ghigo, 2012). Moreover, *B. thetaiotaomicron* targeted *O*-glycan linkages by upregulating fucosidases and galactosidases. This result can be explained by the fact that *B. thetaiotaomicron* will alter its transcriptional response when limited nutrients are available. For example, *B. thetaiotaomicron* in a co-culture will deploy PULs from the associated substrates that are not present in the culture, as a way of finding new substrates that might be available. However, follow-up experiments will be needed to shed light on the transcriptional response of *B. thetaiotaomicron* in a co-culture. This alteration in the glycan metabolism has been previously described, where *B. thetaiotaomicron* shifted from dietary polysaccharides to mucus glycan metabolism in the absence of fiber (Sonnenburg, 2005). Furthermore, it has been described before that *B. thetaiotaomicron* can simultaneously and rapidly respond to multiple glycans (Rogers et al., 2013). In the same study, it is highlighted that the expression of some glycan utilization genes is reduced by the presence of alternative carbohydrates. *B. thetaiotaomicron* in order to increase its fitness in the competitive gut ecosystem, will possibly deploy proteins with multiple starch-binding domains on its surface (Cameron et al., 2012). Accordingly, *A. muciniphila* in response to *B. thetaiotaomicron* GHs expression, upregulated β-galactosidase (GH2), β-hexosaminidase (GH20), and α-L-fucosidase (GH29), targeting *O*-glycans linkages also found in mucin. However, *A. muciniphila* displayed no alterations in its glycan degrading machinery between mono or co-culture conditions, which demonstrates the robustness of the bacterium in a community where the only source of nutrients is mucin glycans. Exploitative competition for nutrients was also observed during the *in vivo* experiment. We observed an overall increase in GHs expression in co-culture conditions from both *A. muciniphila* and *B. thetaiotaomicron*. Since there is no evidence that *A. muciniphila* can utilize dietary fibers, we suggest that two different niches for bacterial colonization were established during the mice colonization.

In the *in vitro* co-culture, *B. thetaiotaomicron* significantly upregulated a cluster of genes encoding hypothetical proteins (BT_2217 – 2221). Three genes of this cluster (BT_2217, BT_2219 and BT_2220) were earlier characterized as C10 protease genes that encode for the C10 family of papain-like cysteine proteases, three of which are genetically clustered, and are associated with two staphostatin-like inhibitors (BT_2218, BT_2221) that may play diverse roles in the interaction with the host (Thornton et al., 2012). Moreover, some studies suggest that cysteine proteases may be located in the outer member vesicles (OMVs), as *B. thetaiotaomicron* preferentially packages lipidated glycosidases and proteases onto OMVs (Hutchings et al., 2009; Elhenawy et al., 2014; Schwechheimer and Kuehn, 2015). These OMVs can interact with host epithelial cells and other bacteria, thus aiming to hydrolyze extracellular host and bacterial targets (Schwechheimer and Kuehn, 2015). Furthermore, it has also been described that cysteine proteases are used from gut bacteria, such as *Porphyromonas gingivalis*, to cleave the mucus proteins in the gut epithelial barrier (van der Post et al., 2013; Vergnolle, 2016). Therefore, in our experiment, the upregulation of cysteine proteases and the associated inhibitors of *B. thetaiotaomicron* in the co-culture *in vitro* could indicate an additional aid to the mucus degradation or a mechanism to prey on or inhibit its competitor—*A. muciniphila*, thus *B. thetaiotaomicron* will share the same nutritional environment.

*In vitro*, the co-culture conditions caused the bacteria to significantly upregulate a cluster (BT_3439–3441) from *B. thetaiotaomicron*, which encodes a gene for membrane attack complex perforin (MACPF) activity (BT_3439) (Xu et al., 2010; Chatzidaki-Livanis et al., 2014). This gene was 60-fold higher expressed (*q* < 0.05) in the co-culture than in the monoculture. *B. thetaiotaomicron* is known to secrete antimicrobial proteins known as Bacteroidales-secreted antimicrobial proteins (BSAPs) (Chatzidaki-Livanis et al., 2014; Roelofs et al., 2016; Shumaker et al., 2019). These BSAPs contain a MACPF domain and target a different class of surface molecule. For example, BSAP-1 and BSAP-4 target the β-barrel outer-membrane protein (OMP), and BSAP-2 and BSAP-3 target the lipopolysaccharide (LPS) glycan (core or *O*-antigen) of the sensitive Bacteroidetes strains that lack the MACPF protein. To date, no study has described the activity of BSAPs against other members of microbial communities, except for Bacteroidetes-sensitive strains. However, in our study, the high fold-change of *B. thetaiotaomicron* MACPF expression in the co-culture may imply that *B. thetaiotaomicron* uses the MACPF to obtain survival advantage in the community. Additionally, for given *Bacteroides species* the MACPF genes are adjacent to the LPS core biosynthesis operon encoding also the replacement glycosyltransferase (GTs) genes of the target strains (McEneany et al., 2018). The MACPF gene (BT_3439), found in our results, is located close to the lipooligosaccharides (LOS) core biosynthesis operon (BT_3362–3385), as it was described before (Jacobson et al., 2018). Our results demonstrated an increase of *B. thetaiotaomicron* GT2 and GT4 expressed in the co-culture compared to monoculture, showing a more than 20.0-fold change and 30.0-fold upregulation, respectively. The acquisition of the MACPF gene occurs with a new set of glycosyltransferase-encoding genes, resulting in an altered LPS glycan structure (Roelofs et al., 2016; McEneany et al., 2018). Therefore, these findings indicate that another type of competition has occurred in the co-culture, where *B. thetaiotaomicron* might attempt to attack *A. muciniphila* through the production of an antimicrobial protein (interference competition). On the other hand, one would expect that *A. muciniphila* abundance in the co-culture would be affected by *B. thetaiotaomicron*'s attempt to reduce its fitness, but this is not what we observed. In co-culture conditions, both *in vitro* and *in vivo, A. muciniphila* significantly upregulated ABC transporters. It has been described before that ABC transporters involved in membrane transport provide antimicrobial peptide resistance (Wang et al., 2016). Another study revealed that the ABC transporter YejABEF from *Brucella melitensis* is essential for its survival and resistance against host antimicrobial proteins. *A. muciniphila* may deploy its ABC transporters that are regulated by the adjacent two-component system-encoding genes to efflux the secreted antimicrobial peptides outside the cell that perhaps are released by *B. thetaiotaomicron*. Furthermore, in the co-culture, *A. muciniphila* upregulated in both the bioreactors (3.2-fold change, *q* < 0.05) and the cecum (2.5-fold change, *q* < 0.05), a gene encoding for *O*-antigen ligase activity (Amuc_0088). This *A. muciniphila O*-antigen was found to be a neighboring gene of the capsular polysaccharide biosynthesis protein (Amuc_2078) (Szklarczyk et al., 2014), which is part of an operon of 20 genes (Amuc_2077 – 2097) predicted to be involved in LPS biosynthesis. The upregulation of *O*-antigen ligase could be used by *A. muciniphila* to counteract *B. thetaiotaomicron*'s antimicrobial peptides. A previous study observed that an *O*-antigen ligase, a Lipid A and 19 other LPS biosynthesis genes were used by *E. coli* to gain resistance to bacteriocins, such as colicin, and other antimicrobial peptides. In these 21 genes, six GTs were found to be significantly upregulated in the presence of colicins, thus providing fitness benefits to uropathogenic *E. coli* (Sharp et al., 2018). In our results, three *A. muciniphila* glycosyl transferases family 4 (Amuc_2088–2090) were above a 2.5-fold change in the co-culture *in vitro*, while, in the cecum, all nine GTs (Amuc_2081–2084, Amuc_2088–2090, Amuc_2093–2094) of this cluster exhibited more than a 1.5-fold difference in the co-culture. Therefore, *A. muciniphila* in a highly competitive environment may be resistant to antimicrobial peptides by using its putative LPS-associated core biosynthesis genes.

In our study, we showed that the presence of *B. thetaiotaomicron* in mice's distal colon and *A. muciniphila* mice's cecum altered the host's response compared to the co-culture. In distal colon co-culture, the host significantly upregulated genes encoding for glycosyltransferase (B3gnt7, St8sia5, Gal3st2c) and sulfotransferase activity (Chst3) involved in protein glycosylation. A recent study observed that more glycosyltransferases, involved in Muc2 O-glycosylation, were detected in the colon than in the small intestine (Kataoka and Huh, 2002). It was observed that B3gnt7, which encodes for β1,3-*N*-acetylglucosaminyltransferase activity, is responsible for elongation of glycans by GlcNAc and together with the B4galt enzymes (B4galt1 and B4galt4) synthesize lactosamine elongations (Arike et al., 2017). Furthermore, B3gnt7 is described to participate in keratan sulfate GAG synthesis (Littlechild et al., 2018). Interestingly, another study highlighted that B3gnt7, together with other transferases, is considered to be responsible for the synthesis of extended sulfated glycans (Lu et al., 2014). Additionally, Chst3 encodes for chondroitin 6-sulfotransferase and catalyzes the transfer of sulfate to position 6 of the *N*-acetylgalactosamine residue of GAG chondroitin sulfate (Uchimura, 1998; Uchimura et al., 2002). Sulfated glycans are abundant in both the small intestine and colon (Arike et al., 2017) and they are also present in the mucosal layer. Therefore, B3gnt7 and Chst3 are used from the host to synthesize keratan and chondroitin sulfate GAGs, which may constitute a source of nutrients for *B. thetaiotaomicron* in the gut. In the cecum, a non-significant increase of genes that encode from transferase activity participating in glycan biosynthesis pathway was also observed. The increased abundance of genes that encode for protein glycosylation in the co-colonized mice could be due to increased bacterial diversity in the gut of the mice. In earlier studies, more glycosyltransferases and longer glycans were detected in the mouse intestine as the intestinal colonization was being increased (Arike et al., 2017). Our data indicate enrichment in the glycosylation process in mice after colonization with glycan-degrading bacteria. This enrichment could constitute a marker for a healthy intestine as abnormal glycosylation was observed in multiple human diseases (Stanley, 2016; Reily et al., 2019).

This study demonstrated that two-glycan degrading bacterial species respond to a nutrient deficit by expressing genes encoding for protein activity related to glycan-degrading enzymes as well as ABC transporters and antimicrobial proteins as a result of their interaction. We showed that when mucin glycans were the only source of nutrients in the environment, *B. thetaiotaomicron* expressed genes involved in antimicrobial activity. Furthermore, our study indicates that *A. muciniphila* was able to sustain its abundance in the presence of *B. thetaiotaomicron* by overexpressing genes that are predicted to be involved in LPS core biosynthesis and some ABC transporters. These results suggest how robust and resilient *A. muciniphila* could be in the human gut, provided its access to mucin glycans is not limited. *A. muciniphila* is known for its capacity to utilize and degrade glycans found on the outer mucosal layer (Derrien et al., 2004). Therefore, the ability of *A. muciniphila* to retain its major function (mucin glycans utilization) and its abundance in a competitive environment implies a colonization advantage of *A. muciniphila* in the gut. Overall, this study gives more insight in how gut microbes adapt to their environment and respond toward host conditions and other microbiota members.

## Materials and Methods

### Bacterial Growth Conditions

Precultures of the references strains of *Akkermansia muciniphila* Muc^T^ (ATTC BAA-835) and *Bacteroides thetaiotaomicron* (VPI-5482) were grown in basal medium as described previously (Derrien et al., 2004). The medium was supplemented with crude hog gastric mucin (0, 5% Type III, Sigma-Aldrich, St. Louis, MO, USA). All the anaerobic bottles were supplemented with 1 % v/v of CaCl_2_ and vitamins mixture solution as described previously (Stams et al., 1993). Incubations were performed in serum bottles sealed with butyl-stoppers at 37°C under anaerobic conditions provided by a gas phase of 182 kPa (1.5 atm) N_2_/CO_2_.

### Anaerobic Continuous Fermentation

*In vitro* fermentations were conducted in three parallel bioreactors (DasGip, Eppendorf, Germany) filled with 250 ml of basal medium as it has been described before (Plugge, 2005) at 37°C, at a controlled pH of 6.5 and at a stirring rate of 150 rpm. The bioreactors and the feed bottle were supplemented with 0.5% of crude mucin, 1% of vitamin solution and at the beginning of the fermentation. Anaerobic conditions were succeeded by sparging the media with N_2_/CO_2_ continuously (6 sL/h). Experiments were performed with 1% (v/v) supplementation of CaCl_2_ and vitamin mixture as described previously. The media in both feed and bioreactors were reduced with 0.05% l-Cysteine-HCl in order to achieve anaerobic conditions. The bioreactors were inoculated with a normalized O.D. of 1.0 of both species to achieve same starting cells density in the beginning of the fermentation. The flow rate of the feed was set at 20 ml/h and the recovery rate of media was 12.5 h. In the end of the fermentation (120 h) samples were taken for total RNA extraction. The growth was measured by spectrophotometer as optical density at 600 nm. The experiment was done in three biological replicates.

### *In vivo* Experiment

The study was conducted in accordance with institutional guidelines for the care and use of laboratory animals. All animal procedures related to the purpose of the research were approved by the University of Gothenburg Animal Studies Committee under the Ethical license of the national competent authority, securing full compliance the European Directive 2010/63/EU for the use of animals for scientific purposes. Female, 10–12 weeks old Swiss Webster germ free (GF) mice (*Mus musculus*), were used in the current study. All mice were obtained from the in-house breeding facility. The GF status of the mice was tested by culturing fecal bacteria anaerobically and aerobically, and also by PCR for bacteria 16S rDNA using the primers 27F and 1492R. One week prior the experiment mice were moved from the breeding isolators to the dedicated experimental isolators. For all colonization experiments, mice were maintained the plastic experimental isolators, in open top cages with dimensions 20 × 14 × 35 cm (incl. metal rack and water bottle) with wooden bedding (Aspen Nesting Material), housed in a climate-controlled room (20 ± 2°C and 60% humidity) and subjected to a strict 12 h light/dark cycle (7:00 a.m. −7:00 p.m.) with free access to autoclaved water and food. Mice were fasted at 9:00 a.m. for 4 h prior gavage (intragastrical) with 0.2 ml of active culture (for both mono- and co-colonization). We have estimated the group size based on previously published studies where GF mice were colonized either with *Akkermansia muciniphila* or *Bacteroides thetaiotaomicron* (Mahowald et al., 2009; Derrien et al., 2011). In all these studies at least 3 mice were included in each group. In our study the smallest group was *n* = 5 mice. For *B. thetaiotaomicron* mono-colonization (*n* = 5 mice) and bi-colonization (*n* = 7 mice) 10^8^ CFU/ml bacterial culture have been used. For *A. muciniphila* (*n* = 8 mice) mono-colonization 10^9^ CFU/ml bacterial culture have been used. For all three colonization, mice were inoculated with a single gavage of the respective culture. The mice received during the 14 day of colonization chow diet (5021 rodent diet, LabDiet; fat 9% wt/wt). At the end of the study mice were euthanized by cervical dislocation. Intestinal segments (cecum and distal colon) were harvested and immediately snap-frozen in liquid nitrogen and stored at −80°C until further processed. Cecum and distal colon content were collected and stored in RNA Later (Sigma) at −20°C until further processed.

### RNA Isolation

Total RNA was isolated for both the bioreactors' samples and mice intestinal segments (cecum and distal colon) by a method combining the Trizol® reagent and the RNeasy Mini kit (QIAGEN, Germany) as described previously (Chomczynski, 1993; Zoetendal et al., 2006). Four microliters of p-mercaptoenthanol and 0.4 ml of buffer RLT were added to 1 ml of Trizol® reagent containing the bacterial pellet or the intestinal segment. The mixture was transferred to a tube containing 0.8 g of glass beads (diameter 0.1 mm), followed by three times of bead beating for 1 min at 5.5 m/s with ice cooling steps in between. Subsequently, 0.2 ml of ice-cold chloroform was added. The solution was mixed gently followed by centrifugation at 12,000 × *g* for 15 min at 4°C. The RNA isolation was continued with the RNA clean-up according to the manufacturer's instructions for the RNeasy Mini kit. Genomic DNA was removed by an on-column DNase digestion step during RNA purification (DNase I recombinant, RNase-free, Roche Diagnostics, Germany). RNA concentration was measured by nanodrop and RNA quality was assessed by Qsep bioanalyzer (BiOptic Inc., Taiwan).

### Transcriptome Sequencing (RNA-Sequencing)

Total RNA samples were further processed by Novogene for RNA-seq.

### Transcriptome Analysis

Illumina reads have been trimmed for low quality and adapters with fastp (v0.20.0) (21) using default settings. rRNA sequences have been removed with bbduk (v38.79) (https://sourceforge.net/projects/bbmap/) using the following parameters *k* = 31 and ref=riboKmers.fa.gz. Transcripts from the reference strain of *A. muciniphila* (GCF_000020225.1) have been quantified with RSEM (v1.3.1) (Li and Dewey, 2011) in combination with bowtie2 (v2.3.5.1) (Langmead and Salzberg, 2012). Mapping and read quality were inspecting using MultiQC. Tximport (v1.12.3) was used to import read-counts obtained from RSEM into Rstudio (Soneson et al., 2016). These imported reads were used for the differential expression analysis using DESeq2 package. 5 (version 2.8.12) was used for RDA and PCA analysis based on the relative abundance data. All further analysis was done using R version 3.6.3 in Rstudio version 1.2.5019. Genes with adjusted *p*-values (*q*-value) < 0.05 and Log2 Fold Change >0.58 were considered significant. The datasets presented in this study are deposited in the European Nucleotide Archive (https://www.ebi.ac.uk/ena), under accession PRJEB41424.

### Genomic DNA Isolation and Quantitative PCR

The abundance of *A. muciniphila* and *B. thetaiotaomicron* in mono- and co-culture was determined by quantitative PCR. Bacterial *in vitro* cultures were harvested 21,000 × g for 15 min. DNA extractions from the *in vitro* samples were performed using MasterPure^TM^ Gram Positive DNA Purification Kit (Epicenter, Lucigen, USA). The genomic DNA from the *in vivo* samples (cecum and distal colon) content was isolated using bead beating. The steps that were followed during DNA isolation from cecum and distal colon are listed in Supplementary Table 10. The DNA concentrations were measured fluorometrically (Quibit dsDNA BR assay; Invitrogen) and adjusted to 1 ng/μl prior to use as the template in qPCR. Primers targeting the 16S rRNA gene of *A. muciniphila* (AM1 5′-CAGCACGTGAAGGTGGGGAC-3′ and AM2 5′-CCTTGCGGTTGGCTTCAGAT-3′; 327 bp and *B. thetaiotaomicron* (BthetaFw 5′-ATA GCCTTTCGAAAGRAAGAT-3′ and BthetaRv 5′-CCAGTATCAACTGCAATTTTA-3′; 500 bp) were used for quantification. Standard curves of both species were prepared with nine standard concentrations from to 10^0^ to 10^8^ gene copies/μl. qPCR was performed in triplicate with iQ SYBR green supermix (Bio-Rad) in a total volume of 13 μl prepared with primers at 500 nM in 384-wells plates with the wells-sealed with optical sealing tape. Amplification was performed with an iCycler (Bio-rad) and the following protocol: one cycle of 95°C for 10 min; 40 cycles of 95°C for 15 s, 60°C for 20s, and 72°C for 30 s each; one cycle of 95°C for 1 min; and a stepwise increase of temperature from 60 to 95°C (at 0.5°C per 5 s) to obtain melt curve data. Data were analyzed using the Bio-Rad CFX Manager 3.0.

### High-Performance Liquid Chromatography

For fermentation product analysis, samples were obtained at different time points of the incubation period. Crotonate was used as the internal standard and the external standards were lactate, formate, acetate, propionate, butyrate, isobutyrate, 1,2-propanediol, sialic acid, and glucose. Substrate conversion and product formation were measured with Shimadzu LC_2030C equipped with a refractive index detector and a Shodex SH1011 column. The oven temperature was set at 45°C with a pump flow of 1.00 mL/min using 0.01N H_2_SO_4_ as eluent. All samples and standards (10 μl injection volume) ran for 20 min. Standards were prepared with concentrations ranging from 2.5 to 25 mM.

### Statistical Analysis

Statistics were performed using student's *t*-test was used and corrected for multiple testing using False Discovery Rate (FDR) correction for multiple corrections. Data are presented as mean ± standard deviation (SD), unless stated otherwise. Adjusted *p* < 0.05 were considered significant.

## Data Availability Statement

The datasets presented in this study can be found in online repositories. The names of the repository/repositories and accession number(s) can be found at: https://www.ebi.ac.uk/ena, PRJEB41424.

## Ethics Statement

The animal study was reviewed and approved by University of Gothenburg Animal Studies Committee.

## Author Contributions

IK and CB designed the experiments. IK performed the *in vitro* experiments and obtained and analyzed all the data with the assistance of SA. PK-D performed the *in vivo* experiment. BN processed the raw RNA-sequencing data through an internal pipeline and deposited and made the data publicly open in an acceptable repository. SA and CB were also involved in the interpretation of the results and wrote the manuscript. FB, JK, and WV supervised the work. CB conceived and supervised this work. All authors have read the manuscript and approved it for submission.

## Conflict of Interest

JK is on the payroll of Nutricia research. The remaining authors declare that the research was conducted in the absence of any commercial or financial relationships that could be construed as a potential conflict of interest.
