# Supplementary material for: A Continuous Battle for Host-Derived Glycans Between a Mucus Specialist and a Glycan Generalist in vitro and in vivo
Source: Front Microbiol. 2021 Jun 24;12:632454. doi: 10.3389/fmicb.2021.632454 (PMC8264420; doi:10.3389/fmicb.2021.632454)
Supplement: Supplementary file 1 [file Data_Sheet_1.PDF]

Supplementary Data

Supplementary Table 1: Bacterial counts (average) of *A. muciniphila* and *B. thetaiotaomicron* in mono-cultures and co-cultures in both *in vitro* and *in vivo* experiments. The standard error of the *in vitro* samples is calculated for three biological samples. The standard error of the *in vivo* samples is calculated based on the number of mice used in each condition (*A. muciniphila* mono-colonization; n=8 mice, *B. thetaiotaomicron* mono-colonization; n=5 mice, bi-colonization; n=7 mice).

	In vitro		*In vivo*			
			Cecum		Distal Colon	
	Cells/ml	***Standard error***	***Cells/ng DNA***	***Standard error***	***Cells/ng DNA***	***Standard error***
A. muciniphila mono-culture	4.41E+07	2.17E+07	3.48E+04	1.08E+04	1.15E+04	3.84E+03
A. muciniphila co-culture	9.78E+07	4.89E+07	1.98E+04	6.80E+03	1.38E+03	5.05E+02
B. thetaiotaomicron mono-culture	2.46E+06	8.72E+05	5.70E+04	1.07E+04	3.31E+03	1.38E+03
B. thetaiotaomicron co-culture	2.12E+06	1.75E+06	1,10E+05	3.74E+04	1.24E+05	6.30E+03

Supplementary Table 2: Fermentation products of *A. muciniphila* and *B. thetaiotaomicron* grown in monocultures and co-culture

	Akkermansia muciniphhila				***Bacteroides thetaiotaomicron***			**Co-culture**			
**Time point (hours)**	**Acetate (mM)**	**Propionate (mM)**	**1,2-propanendiol (mM)**	**Succinate (mM)**	**Acetate (mM)**	**Propionate (mM)**	**1,2-propanendiol (mM)**	**Acetate (mM)**	**Propionate (mM)**	**1,2-propanendiol (mM)**	**Succinate (mM)**
0	0.00 ± 0.00	0.00 ± 0.00	0.00 ± 0.00	0.00 ± 0.00	0.00 ± 0.00	0.00 ± 0.00	0.10 ± 0.21	0.00 ± 0.00	0.00 ± 0.00	0.00 ± 0.00	0.00 ± 0.00
4	0.10 ± 0.20	0.00 ± 0.00	0.05 ± 0.11	0.00 ± 0.00	0.19 ± 0.37	0.00 ± 0.00	0.00 ± 0.00	0.40 ± 0.48	0.00 ± 0.00	0.00 ± 0.00	0.00 ± 0.00
8	1.83 ± 1.42	1.18 ± 1.00	0.07 ± 0.15	0.18 ± 0.21	0.76 ± 0.57	0.00 ± 0.00	0.00 ± 0.00	3.29 ± 4.35	1.18 ± 2.21	0.14 ± 0.17	0.66 ± 1.25
12	7.97 ± 2.66	5.71 ± 2.34	0.49 ± 0.50	0.47 ± 0.56	1.23 ± 0.54	0.18 ± 0.36	0.00 ± 0.00	7.31 ± 5.50	4.47 ± 3.77	0.22 ± 0.18	0.59 ± 0.96
24	10.43 ± 1.95	8.22 ± 1.27	0.69 ± 0.71	0.10 ± 0.19	2.18 ± 0.64	0.00 ± 0.00	0.06 ± 0.12	12.05 ± 1.78	8.24 ± 2.32	0.53 ± 0.61	0.32 ± 0.64
48	10.50 ± 2.16	8.53 ± 1.01	0.78 ± 0.72	0.10 ± 0.20	2.25 ± 0.97	0.65 ± 0.85	0.06 ± 0.11	13.38 ± 1.10	9.39 ± 1.35	0.54 ± 0.63	0.38 ± 0.60
72	9.73 ± 0.73	8.25 ± 0.61	0.71 ± 0.51	0.10 ± 0.20	2.72 ± 1.23	0.67 ± 0.91	0.06 ± 0.12	13.21 ± 0.90	9.87 ± 0.99	0.56 ± 0.55	0.00 ± 0.00
96	9.02 ± 1.30	7.45 ± 0.79	0.54 ± 0.45	0.44 ± 0.65	2.55 ± 1.27	1.07 ± 1.58	0.06 ± 0.13	13.28 ± 0.86	10.07 ± 0.70	0.38 ± 0.32	0.00 ± 0.00
120	9.97 ± 1.20	8.29 ± 0.89	0.59 ± 0.23	0.18 ± 0.21	3.33 ± 0.98	1.25 ± 1.41	0.07 ± 0.15	12.82 ± 1.53	10.36 ± 0.54	0.31 ± 0.25	0.00 ± 0.00

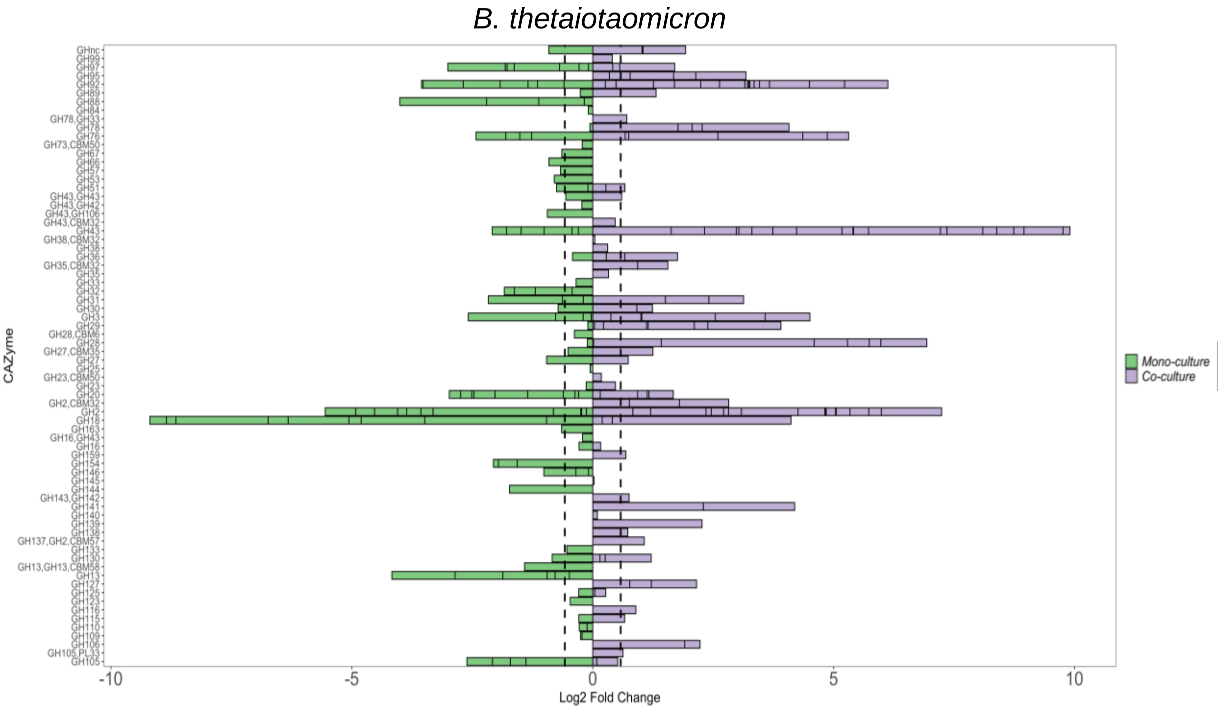

Supplementary Figure 1: Differential expression of *B. thetaiotaomicron* GHs between mono and co-culture during *in vitro* fermentation. Only the CAZymes with adjusted p-value < 0.05 are shown as averages. The grey dotted line indicates Log2 Fold Change threshold (Log2 Fold Change = 0.58)

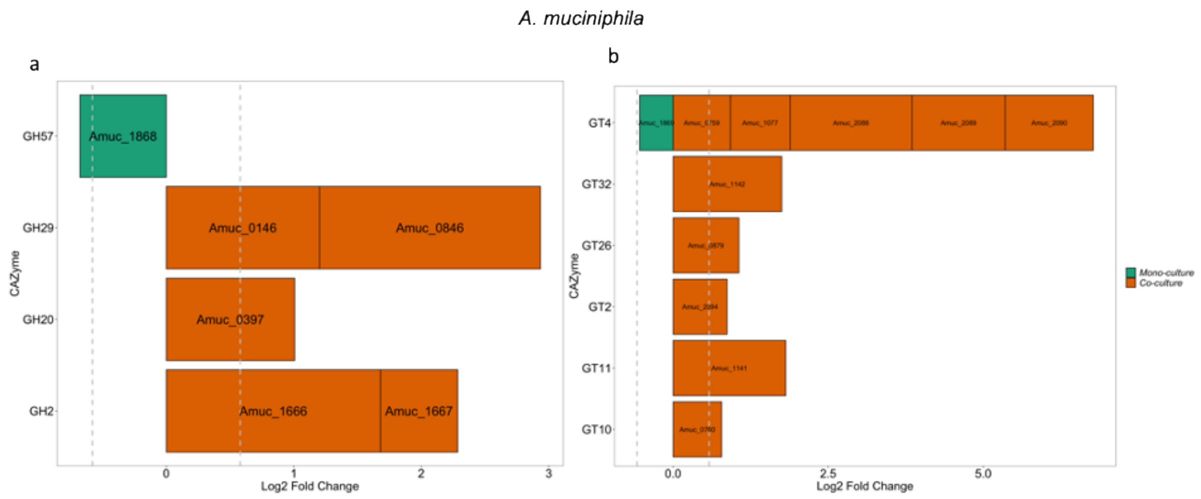

Supplementary Figure 2: Differential expression of GHs and GTs between mono and co-culture during *in vitro* fermentation. a) *A. muciniphila* GHs, b) *A. muciniphila* GTs. Only the CAZymes with adjusted p-value < 0.05 are shown as averages. The grey dotted line indicates Log2 Fold Change threshold (Log2 Fold

Supplementary Table 3: Upregulated *B. thetaiotaomicron* genes belonging to PULs in the co-culture

GeneName	FoldChange	-Log10_padj	ProteinName	CAZy_family	Description	Inducing_Glycan
BT_0145	3,09	1,39	glycosyl hydrolase 43 family protein	GH43	Glycoside Hydrolase Family 43	unknown
BT_0196	7,68	3,96	MFS transporter		putative hexose phosphate transport protein	unknown
BT_0206	5,42	1,66	TonB-dependent receptor		susC-like	host/residual dietary glycans (unknown type)
BT_0207	4,54	1,97	RagB/SusD family nutrient uptake outer membrane protein		susD-like	host/residual dietary glycans (unknown type)
BT_0208	6,04	2,67	DUF4984 domain-containing protein		hypothetical protein	host/residual dietary glycans (unknown type)
BT_0209	3,85	3,27	DUF5003 domain-containing protein		hypothetical protein	host/residual dietary glycans (unknown type)
BT_0210	2,61	2,11	DUF4458 domain-containing protein		leucine-rich repeat protein, function unknown	host/residual dietary glycans (unknown type)
BT_0211	3,64	1,76	BACON domain-containing protein		hypothetical protein	host/residual dietary glycans (unknown type)
BT_0212	2,68	1,67	S8 family serine peptidase		protease	host/residual dietary glycans (unknown type)
BT_0263	4,82	5,20	six-hairpin glycosidase	PL27	hypothetical protein	unknown
BT_0317	2,30	1,43	TonB-dependent receptor		susC-like	mucin O-glycans (N-acetyllactosamine, adult and suckling mouse)
BT_1024	2,95	3,19	RagB/SusD family nutrient uptake outer membrane protein		susD-like	unknown
BT_1025	2,71	2,43	TonB-dependent receptor		susC-like	unknown
BT_2170	2,71	2,43	hypothetical protein		hypothetical protein	host glycans (unknown type, PMG phases 1 and 2)
BT_2171	2,83	3,98	DUF4974 domain-containing protein		anti-sigma factor	host glycans (unknown type, PMG phases 1 and 2)
BT_2172	3,28	3,74	TonB-dependent receptor		susC-like	host glycans (unknown type, PMG phases 1 and 2)
BT_2173	3,44	8,72	DUF4249 domain-containing protein		susD-like	host glycans (unknown type, PMG phases 1 and 2)
BT_2362	3,41	2,67	TonB-dependent receptor		susC-like	unknown
BT_2363	5,26	2,89	RagB/SusD family nutrient uptake outer membrane protein		susD-like	unknown
BT_2393	2,09	1,31	TonB-dependent receptor		susC-like	probable mucin O-glycans (adult and suckling mouse, N-acetyllactosamine)
BT_2529	7,92	1,61	histidinol-phosphatase		hypothetical protein	unknown
BT_2531	4,23	2,93	TonB-dependent receptor		susC-like	unknown
BT_2532	3,91	3,44	RagB/SusD family nutrient uptake outer membrane protein		susD-like	unknown
BT_2859	3,22	1,75	TonB-dependent receptor		susC-like	unknown
BT_2900	2,81	1,50	family 43 glycosylhydrolase	GH43	Glycoside Hydrolase Family 43	unknown
BT_2903	2,72	2,13	DUF4959 domain-containing protein		hypothetical protein	unknown
BT_2955	4,77	2,28	formate acetyltransferase, formate acetyltransferase, formate acetyltransferase		formate acetyltransferase 2	unknown
BT_2956	5,41	1,88	glycyl-radical enzyme activating protein		putative pyruvate formate-lyase 3 activating enzyme	unknown
BT_2968	1,95	1,64	TonB-dependent receptor		susC-like	unknown
BT_2969	2,26	1,72	beta-galactosidase, beta-galactosidase, beta-galactosidase	GH2	Glycoside Hydrolase Family 2	unknown
BT_3016	2,95	1,34	TonB-dependent receptor		TonB-dependent receptor	host glycans (unknown type, PMG phase 2)
BT_3017	5,02	1,67	acid phosphatase		acid phosphatase	host glycans (unknown type, PMG phase 2)
BT_3024	2,70	2,43	TonB-dependent receptor		susC-like	unknown
BT_3048	3,13	3,47	hypothetical protein		hypothetical protein	host glycans (unknown type, adult and suckling mouse)
BT_3176	1,85	1,36	DUF5006 domain-containing protein	GHnc	hypothetical protein	unknown
BT_3278	4,56	2,88	DUF4974 domain-containing protein		anti-sigma factor	unknown
BT_3301	3,60	1,46	alpha-1%2C6-mannanase	GH76	Glycoside Hydrolase Family 76	unknown
BT_3344	4,72	2,21	DUF4973 domain-containing protein		hypothetical protein	unknown
BT_3345	4,02	1,32	RagB/SusD family nutrient uptake outer membrane protein		susD-like	unknown
BT_3346	3,47	1,34	TonB-dependent receptor		susC-like	unknown
BT_3347	3,04	1,34	hypothetical protein		hypothetical protein	unknown
BT_3489	2,28	1,47	arylsulfatase		arylsulfatase B precursor	host/residual dietary glycans (unknown type)
BT_3498	3,53	1,57	DUF4956 domain-containing protein		hypothetical protein	host/residual dietary glycans (unknown type)
BT_3499	4,41	2,39	DUF2490 domain-containing protein		hypothetical protein	host/residual dietary glycans (unknown type)
BT_3500	2,85	1,60	hypothetical protein		hypothetical protein	host/residual dietary glycans (unknown type)
BT_3501	3,39	2,44	alpha-1%2C6-mannanase	GH76	Glycoside Hydrolase Family 76	host/residual dietary glycans (unknown type)
BT_3570	2,54	1,63	tetratricopeptide repeat protein		TPR-repeat-containing proteins	unknown
BT_3608	2,81	1,87	prolyl oligopeptidase family serine peptidase		hypothetical protein	unknown
BT_3670	3,09	2,44	TonB-dependent receptor		susC-like	host/residual dietary glycans (unknown type)
BT_3671	3,05	1,92	RagB/SusD family nutrient uptake outer membrane protein		susD-like	host/residual dietary glycans (unknown type)
BT_3678	2,06	2,63	response regulator		Hybrid two-component system regulator	unknown
BT_3679	3,57	3,86	hypothetical protein		conserved hypothetical protein, with a conserved domain	unknown
BT_3680	3,86	3,87	TonB-dependent receptor		susC-like	unknown
BT_3775	5,06	4,59	hypothetical protein	GT32	hypothetical protein	a-mannan, host N-glycans
BT_3776	5,66	2,29	hypothetical protein	GT32	hypothetical protein	a-mannan, host N-glycans
BT_3777	4,85	2,17	hypothetical protein		hypothetical protein	a-mannan, host N-glycans
BT_3778	3,31	1,41	hypothetical protein		hypothetical protein	a-mannan, host N-glycans
BT_3989	2,49	3,52	hypothetical protein		hypothetical protein	mucin O-glycans
BT_4038	4,89	1,84	RagB/SusD family nutrient uptake outer membrane protein		susD-like	mucin O-glycans
BT_4039	3,85	1,33	TonB-dependent receptor		susC-like	mucin O-glycans
BT_4040	4,36	1,76	DUF1735 domain-containing protein	CBM32	putative galactose oxidase precursor	mucin O-glycans
BT_4076	3,48	2,48	family 78 glycoside hydrolase catalytic domain	GH78	Glycoside Hydrolase Family 78	host glycans (unknown type, PMG phase 2)
BT_4083	2,09	1,38	DUF4361 domain-containing protein		hypothetical protein	host glycans (unknown type, PMG phase 2)
BT_4119	8,80	6,96	pectate lyase	PL1	Polysaccharide Lyase Family 1	host/residual dietary glycans (unknown type)
BT_4120	3,75	3,87	fibronectin type III domain-containing protein		hypothetical protein	host/residual dietary glycans (unknown type)
BT_4121	3,43	4,77	TonB-dependent receptor		susC-like	host/residual dietary glycans (unknown type)
BT_4122	2,27	1,76	RagB/SusD family nutrient uptake outer membrane protein		susD-like	host/residual dietary glycans (unknown type)
BT_4123	9,03	8,01	glycoside hydrolase family 28 protein	GH28	Glycoside Hydrolase Family 28	host/residual dietary glycans (unknown type)
BT_4124	2,19	1,72	hybrid sensor histidine kinase/response regulator transcription factor		Hybrid two-component system regulator	host/residual dietary glycans (unknown type)
BT_4132	5,80	7,45	chitobiase		putative chitobiase	mucin O-glycans
BT_4133	4,02	2,35	DUF4959 domain-containing protein		hypothetical protein	mucin O-glycans
BT_4134	4,67	6,07	RagB/SusD family nutrient uptake outer membrane protein		susD-like	mucin O-glycans
BT_4135	2,48	2,01	TonB-dependent receptor		susC-like	mucin O-glycans
BT_4136	2,86	2,16	alpha-1%2C3/4-fucosidase	GH29	Glycoside Hydrolase Family 29	mucin O-glycans
BT_4157	2,38	1,34	alpha-galactosidase	GH27,CBM35	Glycoside Hydrolase Family 27	unknown
BT_4266	7,02	3,36	hypothetical protein		hypothetical protein	unknown
BT_4267	5,17	2,24	TonB-dependent receptor		susC-like	unknown
BT_4268	4,51	1,60	RagB/SusD family nutrient uptake outer membrane protein		susD-like	unknown
BT_4269	7,04	1,86	DUF4361 domain-containing protein		hypothetical protein	unknown
BT_4270	4,22	2,20	chitobiase		hypothetical protein	unknown
BT_4708	3,11	3,18	SusD/RagB family nutrient-binding outer membrane lipoprotein		susD-like	unknown
BT_4728	4,49	4,47	MFS transporter		putative hexose phosphate transport protein	unknown

Supplementary Table 4: All *A. muciniphila* mucin-degrading genes expressed in the co-culture

GeneName	baseMean	FoldChange	-Log10_padj	CAZy_family	ProteinName
Amuc_1003	211,05	11,56	17,81		alpha/beta hydrolase
Amuc_1934	102,67	6,46	10,23		hypothetical protein
Amuc_0846	429,93	3,33	6,63	GH29	coagulation factor 5/8 type domain-containing protein
Amuc_1666	282,26	3,20	6,16	GH2	glycoside hydrolase family 2, glycoside hydrolase family 2, glycoside hydrolase family 2
Amuc_2040	518,16	2,46	6,38		M3 family peptidase
Amuc_0146	237,52	2,30	2,19	GH29	alpha-L-fucosidase
Amuc_0397	383,03	2,01	3,73	GH20	beta-N-acetylhexosaminidase
Amuc_1801	213,84	1,60	1,50		alpha/beta hydrolase
Amuc_1667	1045,17	1,52	1,42	GH2	hypothetical protein
Amuc_0697	275,41	1,49	1,25	GH43	beta-glucanase
Amuc_0863	955,34	1,31	0,56	GH105	glycosyl hydrolase family protein
Amuc_0698	302,56	1,28	0,70	GH43	beta-glucanase
Amuc_1815	782,73	1,20	0,25	GH20	beta-N-acetylhexosaminidase
Amuc_0953	3294,08	1,16	0,28		hypothetical protein
Amuc_0565	851,57	1,15	0,25		sulfatase, sulfatase
Amuc_1669	754,53	1,12	0,23	GH20	beta-N-acetylhexosaminidase, beta-N-acetylhexosaminidase, beta-N-acetylhexosaminidase, beta-N-acetylhexosaminidase, beta-N-acetylhexosaminidase, beta-N-acetylhexosaminidase
Amuc_1118	606,58	1,12	0,23		sulfatase
Amuc_0491	718,04	1,12	0,17		sulfatase
Amuc_0060	1266,06	1,11	0,15	GH89	alpha-N-acetylglucosaminidase
Amuc_0539	2573,24	1,11	0,19	GH2	hypothetical protein
Amuc_1187	859,89	1,09	0,12	GH27	hypothetical protein, hypothetical protein, hypothetical protein, hypothetical protein
Amuc_1220	1572,60	1,09	0,16	GH89	alpha-N-acetylglucosaminidase
Amuc_1480	956,72	1,08	0,08		D-alanyl-D-alanine carboxypeptidase
Amuc_0187	724,18	1,06	0,08		peptidase M28
Amuc_1655	635,14	1,04	0,05		sulfatase
Amuc_0875	1069,65	1,02	0,02	GH16	beta-glucanase
Amuc_0369	1307,13	1,01	0,01	GH20	beta-N-acetylhexosaminidase, beta-N-acetylhexosaminidase, beta-N-acetylhexosaminidase, beta-N-acetylhexosaminidase, beta-N-acetylhexosaminidase, beta-N-acetylhexosaminidase
Amuc_0121	3358,09	1,01	0,01		arylsulfatase
Amuc_1924	3243,86	0,99	0,01	GH20	beta-N-acetylhexosaminidase
Amuc_0186	1879,86	0,98	0,02	GH95	glycoside hydrolase family protein
Amuc_0482	245,14	0,98	0,02		alpha/beta superfamily hydrolase
Amuc_0392	4308,91	0,96	0,08	GH29	coagulation factor 5/8 type domain-containing protein
Amuc_1120	6458,88	0,95	0,09	GH95	hypothetical protein
Amuc_1438	3395,89	0,91	0,17	CBM51	glycosyl hydrolase family protein
Amuc_0625	2385,62	0,90	0,16	GH33	exo-alpha-sialidase, exo-alpha-sialidase
Amuc_2164	2480,28	0,89	0,18	GH18	hypothetical protein
Amuc_1074	2324,56	0,88	0,30		sulfatase
Amuc_2108	835,61	1,14	0,25	GH16	glycoside hydrolase family protein
Amuc_0771	2097,93	1,15	0,42	GH35	beta-galactosidase, beta-galactosidase, beta-galactosidase
Amuc_0465	347,67	1,16	0,28		M23 family peptidase
Amuc_0670	2026,14	1,16	0,25		Trypsin-like protein serine protease
Amuc_1008	3368,82	1,16	0,32	GH31	alpha-xylosidase
Amuc_2136	8385,18	1,18	0,35	GH20	glycoside hydrolase family 20, glycoside hydrolase family 20, glycoside hydrolase family 20, glycoside hydrolase family 20, glycoside hydrolase family 20, glycoside hydrolase family 20
Amuc_1106	1692,98	1,19	0,59		peptidase M24
Amuc_0824	8657,71	1,21	0,53	GH2	glycoside hydrolase family 2, glycoside hydrolase family 2, glycoside hydrolase family 2
Amuc_1631	2757,90	1,22	0,66		tail-specific protease
Amuc_1032	2338,78	1,22	0,50	GH20	hypothetical protein
Amuc_0253	581,32	1,25	0,34		M23 family peptidase
Amuc_1686	3086,01	1,31	0,73	GH35,CBM32	beta-galactosidase
Amuc_1755	3031,82	1,35	1,02		sulfatase
Amuc_0176	1116,24	1,36	1,37		hypothetical protein
Amuc_0391	137,88	1,37	0,51		M23 family peptidase

Supplementary Table 5: Upregulated *B. thetaiotaomicron* CAZymes (Log2 fold change >0.58) in the co-culture. The differentially expressed genes (q-value <0.05, Log2 fold change >0.58) are indicated with orange color.

GeneName	baseMean	log2FoldChange	FoldChange	padj	-Log10_padj	ProteinName	CAZy_family
BT_4040	2816,71	2,12	4,36	0,02	1,76	DUF1735 domain-containing protein	CBM32
BT_0865	10720,44	2,62	6,14	0,30	0,52	DUF1735 domain-containing protein	CBM32
BT_1775	57,01	0,68	1,60	0,35	0,45	DUF5010 domain-containing protein	CBMnc
BT_2963	126,26	0,63	1,55	0,14	0,85	glycoside hydrolase family 88 protein	GH105,PL33
BT_0986	337,83	1,91	3,75	0,00	5,06	DNA-binding protein	GH106
BT_3514	203,07	0,66	1,58	0,11	0,97	hypothetical protein	GH115
BT_2908	149,05	0,89	1,86	0,10	1,02	hypothetical protein	GH116
BT_3674	97,20	0,94	1,92	0,05	1,27	glycoside hydrolase family 127 protein	GH127
BT_1003	91,64	0,77	1,70	0,15	0,83	glycoside hydrolase family 127 protein	GH127
BT_4094	169,57	0,96	1,94	0,13	0,90	hypothetical protein	GH130
BT_0996	118,15	1,07	2,10	0,01	2,17	DUF4982 domain-containing protein	GH137,GH2,CBM57
BT_0997	67,40	0,73	1,66	0,19	0,72	hypothetical protein	GH138
BT_0984	61,71	2,27	4,82	0,00	6,06	hypothetical protein	GH139
BT_0338	34,54	2,30	4,91	0,00	2,48	right-handed parallel beta-helix repeat-containing protein	GH141
BT_1002	28,13	1,90	3,72	0,01	2,24	right-handed parallel beta-helix repeat-containing protein	GH141
BT_1020	99,48	0,76	1,69	0,11	0,95	hypothetical protein	GH143,GH142
BT_2901	81,26	0,69	1,61	0,23	0,64	glycosyl hydrolase family 43	GH159
BT_3050	14,96	3,71	13,08	0,00	3,33	glycoside hydrolase family 18 protein	GH18
BT_2969	58,80	1,18	2,26	0,02	1,72	beta-galactosidase, beta-galactosidase, beta-galactosidase	GH2
BT_0993	73,79	1,15	2,22	0,02	1,62	DUF4981 domain-containing protein, DUF4981 domain-containing protein, DUF4981 domain-containing protein	GH2
BT_4684	424,26	1,25	2,38	0,05	1,34	beta-galactosidase, beta-galactosidase, beta-galactosidase	GH2
BT_0983	79,97	0,83	1,78	0,16	0,80	beta-galactosidase, beta-galactosidase, beta-galactosidase	GH2
BT_4074	360,36	1,02	2,03	0,06	1,24	beta-mannosidase	GH2,CBM32
BT_2855	84,36	1,04	2,06	0,06	1,23	beta-mannosidase	GH2,CBM32
BT_2432	233,35	0,76	1,69	0,23	0,64	beta-mannosidase	GH2,CBM32
BT_1621	76,18	0,77	1,71	0,11	0,96	beta-N-acetylhexosaminidase	GH20
BT_3065	86,08	0,74	1,67	0,11	0,97	alpha-galactosidase, alpha-galactosidase, alpha-galactosidase, alpha-galactosidase	GH27
BT_4157	39,16	1,25	2,38	0,05	1,34	alpha-galactosidase	GH27,CBM35
BT_4123	40,80	3,17	9,03	0,00	8,01	glycoside hydrolase family 28 protein	GH28
BT_2378	45,45	1,41	2,65	0,02	1,63	endopolygalacturonase	GH28
BT_4187	180,43	0,96	1,94	0,25	0,60	glycoside hydrolase family 28 protein	GH28
BT_4146	43,64	0,69	1,62	0,35	0,46	glycoside hydrolase family 28 protein	GH28
BT_4136	72,29	1,52	2,86	0,01	2,16	alpha-1%2C3/4-fucosidase	GH29
BT_2970	71,96	0,89	1,86	0,12	0,91	alpha-L-fucosidase	GH29
BT_3798	66,64	0,96	1,94	0,17	0,78	alpha-L-fucosidase	GH29
BT_3009	168,70	1,53	2,88	0,00	2,70	beta-glucosidase, beta-glucosidase, beta-glucosidase	GH3
BT_4714	165,13	0,93	1,90	0,07	1,15	beta-glucosidase, beta-glucosidase, beta-glucosidase	GH3
BT_3300	49,40	1,04	2,05	0,08	1,11	beta-glucosidase, beta-glucosidase, beta-glucosidase	GH3
BT_1780	78,62	0,62	1,54	0,30	0,53	beta-glucosidase, beta-glucosidase, beta-glucosidase	GH3
BT_3026	73,03	0,92	1,89	0,06	1,20	glycosyl hydrolase	GH30
BT_0339	47,44	1,51	2,84	0,02	1,73	alpha-xylosidase	GH31
BT_3299	62,26	0,90	1,87	0,14	0,86	DUF5110 domain-containing protein, DUF5110 domain-containing protein	GH31
BT_3659	172,08	0,72	1,65	0,16	0,81	DUF5110 domain-containing protein	GH31
BT_3654	286,63	0,63	1,54	0,10	1,00	beta-galactosidase	GH35,CBM32
BT_0290	210,64	0,93	1,91	0,20	0,70	beta-galactosidase	GH35,CBM32
BT_3797	150,15	1,09	2,14	0,00	2,31	glycoside hydrolase family 36	GH36
BT_2900	31,29	1,49	2,81	0,03	1,50	family 43 glycosylhydrolase	GH43
BT_0145	27,67	1,63	3,09	0,04	1,39	glycosyl hydrolase 43 family protein	GH43
BT_4095	209,81	0,81	1,75	0,15	0,81	family 43 glycosylhydrolase	GH43
BT_2112	41,19	0,94	1,92	0,27	0,56	family 43 glycosylhydrolase	GH43
BT_3655	57,47	0,74	1,67	0,33	0,48	glycoside hydrolase family 43 protein	GH43
BT_0264	59,41	0,69	1,62	0,55	0,26	family 43 glycosylhydrolase	GH43
BT_0265	41,04	0,66	1,58	0,60	0,22	family 43 glycosylhydrolase	GH43
BT_3656	101,23	0,60	1,52	0,33	0,49	family 43 glycosylhydrolase	GH43,GH43
BT_3501	33,60	1,76	3,39	0,00	2,44	alpha-1%2C6-mannanase	GH76
BT_3301	24,71	1,85	3,60	0,03	1,46	alpha-1%2C6-mannanase	GH76
BT_1883	243,70	0,67	1,60	0,07	1,16	glycoside hydrolase family 76 protein	GH76
BT_1001	43,57	1,77	3,41	0,00	3,42	family 78 glycoside hydrolase catalytic domain	GH78
BT_4076	128,45	1,80	3,48	0,00	2,48	family 78 glycoside hydrolase catalytic domain	GH78
BT_1013	104,16	0,71	1,63	0,12	0,92	family 78 glycoside hydrolase catalytic domain	GH78,GH33
BT_4359	362,97	0,74	1,68	0,12	0,93	alpha-N-acetylglucosaminidase	GH89
BT_4092	193,92	0,73	1,66	0,16	0,79	glycoside hydrolase family 92 protein	GH92
BT_4093	220,39	0,90	1,87	0,19	0,72	glycoside hydrolase family 92 protein	GH92
BT_4073	229,32	0,83	1,78	0,21	0,68	glycoside hydrolase family 92 protein	GH92
BT_2111	173,06	0,78	1,71	0,32	0,49	hypothetical protein	GH92
BT_4682	784,85	1,04	2,06	0,10	0,98	hypothetical protein	GH95
BT_3155	274,60	0,90	1,87	0,26	0,58	glycoside hydrolase family 95 protein	GH95
BT_4581	303,18	1,14	2,21	0,11	0,97	glycoside hydrolase family 97 protein	GH97
BT_3176	75,63	0,88	1,85	0,04	1,36	DUF5006 domain-containing protein	GHnc
BT_2113	176,67	1,02	2,03	0,05	1,29	hypothetical protein	GHnc
BT_0235	35,08	1,33	2,52	0,04	1,38	glycosyl transferase	GT1
BT_1179	549,27	0,63	1,55	0,12	0,92	SP_1767 family glycosyltransferase	GT101
BT_2870	22,25	3,30	9,86	0,00	4,20	glycosyl transferase	GT14
BT_2871	19,09	1,20	2,29	0,20	0,70	glycosyl transferase	GT14
BT_2882	28,09	2,86	7,24	0,00	3,99	glycosyltransferase	GT2
BT_2868	25,19	2,95	7,73	0,00	3,52	glycosyltransferase family 2 protein	GT2
BT_1646	26,60	2,65	6,26	0,00	3,11	glycosyltransferase family 2 protein	GT2
BT_1648	31,65	1,95	3,85	0,00	2,82	glycosyltransferase family 2 protein	GT2
BT_0473	19,33	2,46	5,51	0,00	2,81	glycosyltransferase family 2 protein	GT2
BT_1709	5,49	4,86	29,03	0,01	2,01	glycosyltransferase	GT2
BT_1166	302,13	0,91	1,88	0,01	1,94	glycosyltransferase family 2 protein	GT2
BT_1167	299,41	0,88	1,84	0,04	1,43	glycosyltransferase family 2 protein	GT2
BT_2874	12,62	1,93	3,81	0,06	1,19	glycosyltransferase	GT2
BT_1181	274,41	0,74	1,67	0,08	1,10	glycosyltransferase family 2 protein	GT2
BT_2876	16,24	1,52	2,86	0,09	1,03	glycosyltransferase	GT2
BT_4281	280,45	2,70	6,48	0,24	0,61	glycosyltransferase family 2 protein	GT2
BT_0050	57,90	1,32	2,49	0,26	0,59	glycosyltransferase family 2 protein	GT2
BT_1645	8,12	1,68	3,20	0,28	0,55	glycosyltransferase	GT2
BT_0051	50,84	0,72	1,65	0,56	0,25	glycosyltransferase family 2 protein	GT2
BT_1649	36,00	1,00	2,00	0,23	0,64	glycosyltransferase family 25 protein	GT25
BT_3775	53,50	2,34	5,06	0,00	4,59	hypothetical protein	GT32
BT_3776	61,75	2,50	5,66	0,01	2,29	hypothetical protein	GT32
BT_2864	19,53	5,28	38,88	0,00	4,85	glycosyltransferase	GT4
BT_1712	23,78	2,23	4,70	0,01	1,89	glycosyltransferase family 4 protein	GT4
BT_4282	573,85	2,47	5,55	0,01	1,84	glycosyltransferase family 4 protein	GT4
BT_0478	30,42	1,23	2,34	0,08	1,10	glycosyltransferase family 4 protein	GT4
BT_0392	187,37	1,54	2,90	0,12	0,94	glycosyltransferase family 4 protein	GT4
BT_1180	597,78	0,67	1,59	0,14	0,86	glycosyltransferase family 4 protein	GT4
BT_0610	178,44	1,45	2,74	0,15	0,83	glycosyltransferase family 4 protein	GT4
BT_2866	35,12	0,79	1,73	0,23	0,65	glycosyltransferase family 4 protein	GT4
BT_0394	249,34	1,25	2,38	0,23	0,64	glycosyltransferase family 4 protein	GT4
BT_0391	187,22	1,26	2,39	0,25	0,61	glycosyltransferase	GT4
BT_0608	206,45	1,04	2,06	0,28	0,55	glycosyltransferase family 4 protein	GT4
BT_0609	86,38	0,76	1,70	0,44	0,36	glycosyltransferase	GT4
BT_2873	22,56	1,21	2,32	0,12	0,92	glycosyltransferase family 8 protein	GT8
BT_2865	28,86	3,27	9,63	0,00	5,85	glycosyltransferase family 4 protein	GTnc
BT_4280	534,25	3,14	8,80	0,10	1,01	glycosyltransferase	GTnc
BT_0471	20,17	1,43	2,70	0,12	0,91	glycosyltransferase family 4 protein	GTnc
BT_1175	523,73	0,75	1,68	0,14	0,85	hypothetical protein	GTnc
BT_0522	404,00	0,63	1,54	0,39	0,41	membrane protein	GTnc
BT_4119	37,96	3,14	8,80	0,00	6,96	pectate lyase	PL1
BT_4115	67,56	0,64	1,56	0,32	0,50	pectate lyase	PL1
BT_2254	152,58	1,20	2,30	0,02	1,80	pectate lyase	PL10
BT_0263	67,43	2,27	4,82	0,00	5,20	six-hairpin glycosidase	PL27
BT_4183	75,42	0,76	1,70	0,17	0,77	pectate lyase	PL9

Supplementary Table 5: Upregulated *A. muciniphila* CAZymes (Log2 fold change >0.58) in the co-culture. The differentially expressed genes (q-value <0.05, Log2 fold change >0.58) are indicated with orange color.

GeneName	baseMean	log2FoldChange	FoldChange	padj	-Log10_padj	ProteinName	CAZy_family
Amuc_1666	282,26	1,68	3,20	0,00	6,16	glycoside hydrolase family 2, glycoside hydrolase family 2, glycoside hydrolase family 2	GH2
Amuc_1667	1045,17	0,61	1,52	0,04	1,42	hypothetical protein	GH2
Amuc_0397	383,03	1,01	2,01	0,00	3,73	beta-N-acetylhexosaminidase	GH20
Amuc_0846	429,93	1,73	3,33	0,00	6,63	coagulation factor 5/8 type domain-containing protein	GH29
Amuc_0146	237,52	1,20	2,30	0,01	2,19	alpha-L-fucosidase	GH29
Amuc_0760	151,87	0,78	1,72	0,03	1,54	hypothetical protein	GT10
Amuc_1141	255,26	1,82	3,52	0,00	7,67	alpha-1%2C2-fucosyltransferase	GT11
Amuc_2094	340,68	0,87	1,83	0,01	1,92	hypothetical protein	GT2
Amuc_2093	187,63	0,64	1,55	0,09	1,04	glycosyltransferase family 2 protein	GT2
Amuc_0879	253,73	1,06	2,08	0,00	4,21	glycosyltransferase	GT26
Amuc_1142	639,17	1,75	3,36	0,00	5,52	glycosyl transferase family protein	GT32
Amuc_2088	171,54	1,97	3,91	0,00	9,24	glycosyl transferase family 1	GT4
Amuc_2089	408,77	1,50	2,83	0,00	6,15	group 1 glycosyl transferase	GT4
Amuc_0759	477,85	0,92	1,90	0,00	4,80	hypothetical protein	GT4
Amuc_2090	276,71	1,42	2,68	0,00	4,55	glycosyltransferase family 4 protein	GT4
Amuc_1077	465,70	0,96	1,95	0,00	3,93	glycosyltransferase family 1 protein	GT4

Supplementary Table 7: Top 50 most abundant *B. thetaiotaomicron* genes expressed in the co-culture

GeneName	KO	FoldChange	-Log10_padj	CDS	ProteinName	Metabolic process	Pathway
BT_0016		138,88	5,03	cds15	hypothetical protein		
BT_2221		119,04	15,70	cds2220	hypothetical protein		
BT_2220		101,87	14,60	cds2219	hypothetical protein		
BT_3440		96,05	22,30	cds3438	hypothetical protein		
BT_3441		83,82	19,80	cds3439	hypothetical protein		
BT_1132		78,51	3,53	cds1131	hypothetical protein		
BT_2645		72,16	3,82	cds2644	hypothetical protein		
BT_2641		71,70	3,33	cds2640	hypothetical protein		
BT_2219		70,42	12,60	cds2218	hypothetical protein		
BT_2612		67,67	3,00	cds2611	hypothetical protein		
BT_0020		60,07	2,94	cds19	MACPF domain containing protein		
BT_3439		59,34	21,90	cds3437	hypothetical protein		
BT_1991		59,05	2,67	cds1990	N-acetylmuramoyl-L-alanine amidase		
BT_2312		58,56	2,85	cds2311	hypothetical protein		
BT_4433		51,86	4,42	cds4430	hypothetical protein		
BT_2218		50,27	9,66	cds2217	hypothetical protein		
BT_2960		47,96	3,00	cds2958	hypothetical protein		
BT_2864	K12995	47,02	5,71	cds2862	lipopolysaccharide biosynthesis glycosyltransferase		
BT_1990		46,63	3,02	cds1989	hypothetical protein		
BT_0018		46,05	5,80	cds17	hypothetical protein		
BT_1589		44,87	2,24	cds1588	hypothetical protein		
BT_4023		41,68	2,08	cds4020	transposase		
BT_2996		39,73	2,75	cds2994	hypothetical protein		
BT_4780		39,70	3,12	cds4777	conjugate transposon protein		
BT_4774		37,30	5,14	cds4771	conjugate transposon protein		
BT_4735		37,17	3,49	cds4732	hypothetical protein		
BT_2217		37,07	9,84	cds2216	hypothetical protein		
BT_2652		36,14	1,83	cds2651	hypothetical protein		
BT_2594		31,82	4,30	cds2593	conjugate transposon protein		
BT_1515		30,49	4,43	cds1514	hypothetical protein		
BT_0469		29,78	4,77	cds468	hypothetical protein		
BT_4748		29,57	4,77	cds4745	hypothetical protein		
BT_2131		29,02	8,59	cds2130	hypothetical protein		
BT_0019		26,93	1,31	cds18	hypothetical protein		
BT_1709		25,79	2,44	cds1708	glycosyltransferase		
BT_0538		24,91	1,33	cds537	hypothetical protein		
BT_4435		24,26	4,11	cds4432	hypothetical protein		
BT_4489		23,92	1,72	cds4486	hypothetical protein		
BT_4024		23,25	1,30	cds4021	hypothetical protein		
BT_4491		23,15	1,68	cds4488	hypothetical protein		
BT_1951	K02015	22,67	3,48	cds1950	iron ABC transporter permease		
BT_4490		20,28	1,30	cds4487	hypothetical protein		
BT_2881	K00991	19,20	6,56	cds2879	2-C-methyl-D-erythritol 4-phosphate cytidylyltransferase	Metabolism of terpenoids and polyketides	Terpenoid backbone biosynthesis
BT_0294		18,97	8,55	cds293	hypothetical protein		
BT_3502		17,91	2,35	cds3500	hypothetical protein		
BT_0949		17,62	1,90	cds948	hypothetical protein		
BT_4021		17,34	5,07	cds4018	integrase		
BT_0099		17,08	1,41	cds98	hypothetical protein		
BT_2643		16,22	2,22	cds2642	hypothetical protein		
BT_2696		16,16	1,78	cds2694	hypothetical protein		

Supplementary Table 8: Top 50 most abundant *A. muciniphila* genes expressed in the co-culture

GeneName	KO	FoldChange	-Log10_padj	ProteinName	Metabolic process	Pathway
Amuc_1415		15,43	11,28	hypothetical protein		
Amuc_0892		10,77	12,10	autotransporter domain-containing protein		
Amuc_0388		10,35	1,92	hypothetical protein		
Amuc_1305	K02037	10,03	10,80	ABC transporter permease	Membrane transport	ABC transporters
Amuc_1003	K06889	9,88	13,58	alpha/beta hydrolase		
Amuc_1721		9,49	4,03	hypothetical protein		
Amuc_1303	K02036	9,43	7,48	phosphate ABC transporter ATP-binding protein	Membrane transport	ABC transporters
Amuc_0387		9,22	11,52	hypothetical protein		
AMUC_RS12735		8,25	2,92	hypothetical protein		
Amuc_1304	K02038	8,11	9,75	phosphate ABC transporter%2C permease protein PstA	Membrane transport	ABC transporters
Amuc_0288	K03179	7,92	10,80	hypothetical protein	Metabolism of cofactors and vitamins	Ubiquinone and other terpenoid-quinone biosynthesis
Amuc_1675		7,73	9,38	site-specific integrase		
AMUC_RS12705		7,35	15,93	hypothetical protein		
Amuc_1931	K02015	7,29	8,01	iron ABC transporter permease	signaling and cellular processes	
Amuc_1357		7,11	6,18	hypothetical protein		
Amuc_1083		6,84	4,94	heavy metal translocating P-type ATPase		
Amuc_0854	K03530	6,72	5,57	DNA-binding protein		
AMUC_RS05395		6,48	7,04	alpha/beta hydrolase		
AMUC_RS12525		5,74	1,72	hypothetical protein		
Amuc_1934		5,64	7,16	hypothetical protein		
Amuc_0107		5,64	15,94	hydrophobe/amphiphile efflux-1 family RND transporter		
Amuc_0108		5,41	9,97	efflux RND transporter periplasmic adaptor subunit		
AMUC_RS11805		5,31	4,61	hypothetical protein		
Amuc_1926		5,20	9,90	cytochrome c assembly protein		
Amuc_1387		5,17	13,24	hypothetical protein		
Amuc_1878	K07032	5,16	7,70	lactoylglutathione lyase		
Amuc_1356		5,08	4,25	hypothetical protein		
Amuc_0936		5,06	11,15	hypothetical protein		
AMUC_RS12660		5,01	4,92	glycosyltransferase		
Amuc_1301	K01738	4,98	4,80	cysteine synthase A	Energy metabolism, Amino acid metabolism	Sulfur metabolism, Cysteine and methionine metabolism
Amuc_2091		4,98	9,77	hypothetical protein		
Amuc_1872	K00185	4,95	10,24	polysulfide reductase NrfD	Energy metabolism	Sulfur metabolism
Amuc_1677		4,93	7,43	type II restriction endonuclease		
Amuc_0843		4,84	15,94	hypothetical protein		
AMUC_RS01550		4,75	1,67	hypothetical protein		
Amuc_1930	K02015	4,70	4,24	transporter permease		
Amuc_1160		4,69	9,70	aldo/keto reductase		
Amuc_0386		4,63	5,28	hypothetical protein		
AMUC_RS12600		4,60	3,59	hypothetical protein		
Amuc_1711		4,56	4,22	hypothetical protein		
Amuc_1457	K01814	4,52	10,09	phosphoribosylformimino-5-aminoimidazole carboxamid	Amino acid metabolism	Histidine metabolism
Amuc_1468		4,44	6,35	alpha/beta hydrolase		
AMUC_RS03120		4,43	9,68	ComF family protein		
Amuc_0848	K02238	4,40	12,50	hypothetical protein		
Amuc_1571		4,36	13,58	tRNA (adenosine(37)-N6)-threonylcarbamoyltransferase complex dimerization subunit type 1 TsaB		
AMUC_RS12515		4,35	1,60	hypothetical protein		
Amuc_1351		4,33	2,50	hypothetical protein		
Amuc_1306	K02040	4,30	5,55	phosphate-binding protein	Membrane transport, Signal transduction	ABC transporters , Two-component system
Amuc_0021	K03092	4,26	9,56	RNA polymerase sigma-54 factor	Signal transduction	Two-component system
Amuc_1386	K00796	4,10	10,23	dihydropteroate synthase	Metabolism of cofactors and vitamins	Folate biosynthesis

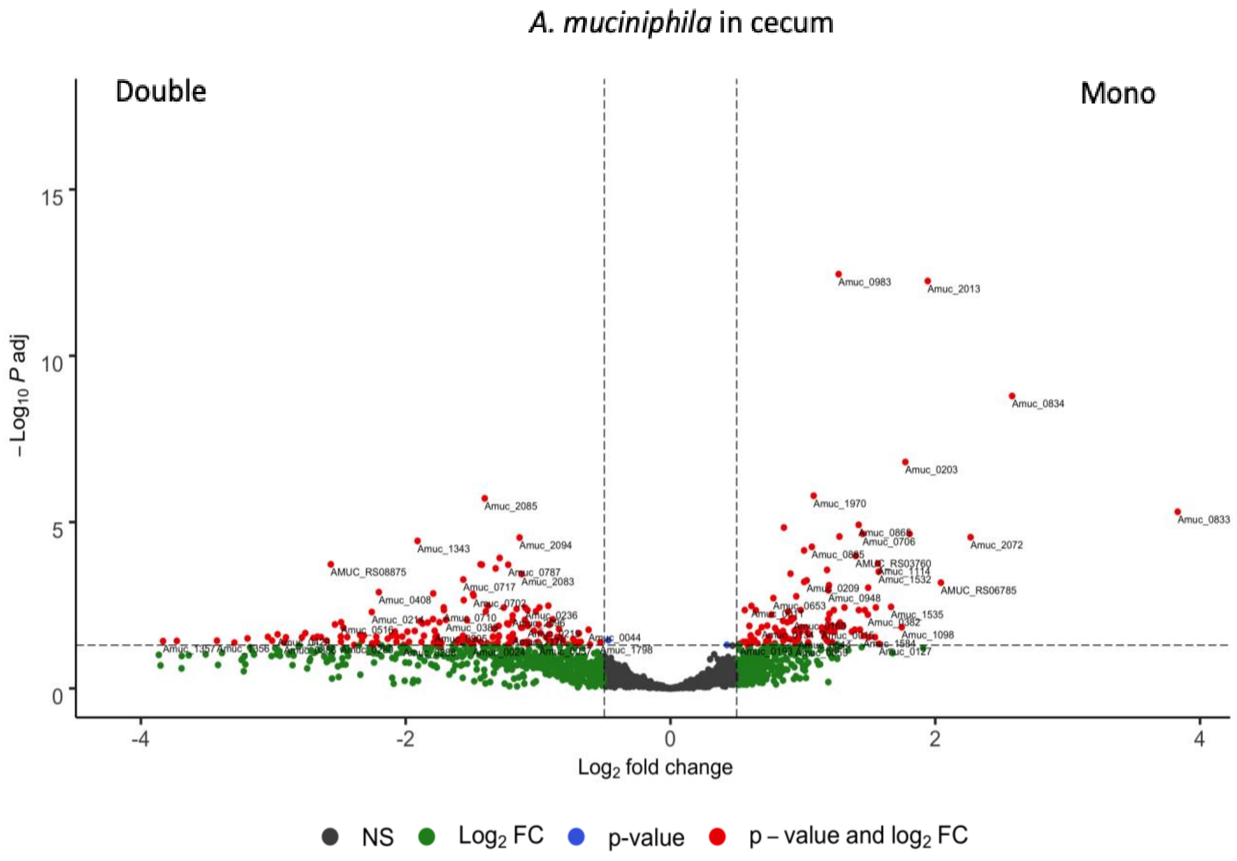

Supplementary Figure 3: Differential expressed genes of *A. muciniphila* between mono- and co-culture in mice cecum. a) Volcano plot of the differential expressed A. muciniphila genes of mono- versus co-culture. Positive Log2 fold change indicates upregulation in co-culture. The horizontal dashed black line adjusted p-value threshold (q-value = 0.05). The vertical dashed black lines indicate Log2 fold change threshold (Log2 fold change = 0.58).

Supplementary Table 9: The most significantly affected host (mouse) genes by the presence of glycan -degraders in cecum. In light blue the 10 most abundant host genes are highlighted.

GeneName	baseMean	log2FoldChange	FoldChange	-Log10-padj	Gene_function
Zzef1-2	58,18	-23,38	10952966,60	7,74	Zinc finger ZZ-type and EF-hand domain-containing protein 1
Cckar	18,00	-8,03	262,04	1,57	cholecystokinin A receptor
Zfp987-2	20,74	-6,81	112,10	1,43	Zinc finger protein 987
Epha6	78,04	-4,82	28,16	1,30	Eph receptor A6
Gjb4	46,46	-3,76	13,56	1,37	gap junction protein%2C beta 4
Cyp4f15	32,87	-2,64	6,25	3,87	cytochrome P450%2C family 4%2C subfamily f%2C polypeptide 15
Insl5	188,08	-2,31	4,94	4,42	insulin-like 5
Mzb1	229,03	-2,07	4,20	6,02	marginal zone B and B1 cell-specific protein 1
Tnfrsf13b	114,56	-1,71	3,28	1,85	tumor necrosis factor receptor superfamily%2C member 13b
Ttll9	47,32	-1,69	3,23	1,57	tubulin tyrosine ligase-like family%2C member 9
Acaa1b	379,23	-1,41	2,65	1,40	acetyl-Coenzyme A acyltransferase 1B
Aqp8	11638,95	-1,39	2,62	4,83	aquaporin 8
Cmtm8	212,97	-1,24	2,37	3,87	CKLF-like MARVEL transmembrane domain containing 8
Tsku	2330,62	-1,24	2,37	1,54	tsukushi%2C small leucine rich proteoglycan
Tmem171	1053,34	-1,12	2,17	1,59	transmembrane protein 171
Pla2g12b	294,90	-1,11	2,16	1,66	phospholipase A2%2C group XIIB
Sdc1	4913,62	-1,04	2,06	2,21	syndecan 1
Kyat1	380,50	-1,00	2,00	1,64	cysteine conjugate-beta lyase 1
Cgref1	575,42	-0,93	1,90	2,41	cell growth regulator with EF hand domain 1
Adipor2	4466,36	-0,70	1,62	1,40	adiponectin receptor 2
Slc2a1	3890,48	-0,63	1,55	1,40	solute carrier family 2 (facilitated glucose transporter)%2C member 1
Prdx5	2341,53	-0,63	1,55	1,82	peroxiredoxin 5
Lgals3	26565,23	-0,62	1,54	1,44	lectin%2C galactose binding%2C soluble 3

Supplementary Table 10: DNA isolation protocol from biopsy sample (cecum and distal colon).

DNA isolation from biopsies	
1)	Add 940µl TE buffer to the biopsy.
2)	Add 50 µl SDS (10%) and 10 µl Proteinase K (20 mg/ml) to 1 ml sample.
3)	Incubate at 55 °C for 1 hour.
4)	Pipet sample to a 2ml bead beating tube containing glass beads (RBB protocol).
5)	Add 150 µl buffered phenol (pH 7-8).
6)	Bead beating for 3 min. at 5,000 rpm. Cool on ice.
7)	Add 150 µl Choroform/isoamylalcohol.
8)	Mix well and centrifuge at maximum speed for 10 minutes at 4ºC.
9)	Remove upper layer and put it in a new tube.
10)	Add 150 µl buffered phenol and 150 µl chloroform/isoamylalcohol, mix and centrifuge at maximum speed for 10 minutes at 4ºC.
11)	Remove upper layer and put in a new tube.
12)	Repeat steps 9) to 10) until the interface of the two layers is clean.
13)	Add 300 µl Choroform/isoamylalcohol (24:1), mix, and centrifuge at maximum speed for 10 minutes at 4ºC.
14)	Remove upper layer.
15)	Add 1 µl 20 mg/ml glycogen and mix the content (important for precipitation of DNA).
16)	Add 1 volume 2-propanol and 1/10 volume of 3M NaAc.
17)	Store at -20°C for 30 minutes.
18)	Centrifuge for 20 minutes at maximum speed.
19)	Remove supernatant and add 500 µl 70% ethanol.
20)	Centrifuge for 5 minutes at maximum speed.
21)	Dry pellet for 15 minuntes.
22)	Rehydrate in 100 µl TE (pH 8.0).
23)	Measure DNA concentration.
